# Low-Frequency Vibrational Spectroscopy and Quantum Mechanical Simulations of the Crystalline Polymorphs of the Antiviral Drug Ribavirin

**DOI:** 10.1021/acs.molpharmaceut.2c00509

**Published:** 2022-08-11

**Authors:** Margaret P. Davis, Timothy M. Korter

**Affiliations:** Department of Chemistry, Syracuse University, 1-133 Center for Science and Technology, Syracuse, New York 13244-4100, United States

**Keywords:** far-infrared spectroscopy, phase transition, crystallography, periodic boundary conditions, computational chemistry

## Abstract

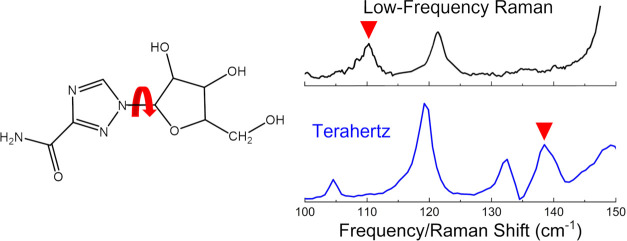

Crystal polymorphism is a common phenomenon in pharmaceutical solids and a critical issue when considering the formulation of therapeutics since multiple polymorphs may form during drug manufacturing. Low-frequency vibrational spectroscopy is sensitive to polymorphic content, and in this work, terahertz time-domain spectroscopy and low-frequency Raman spectroscopy were utilized in the study of crystalline ribavirin, a widely applicable antiviral. Characteristic spectra with numerous peaks in the sub-200 cm^–1^ region were obtained of the more common polymorph of ribavirin (Form II). Solid-state density functional theory (ss-DFT) simulations were then used to optimize the crystal structure of this polymorph and calculate the frequencies and spectral intensities of the lattice vibrations in the low-frequency region. The near-harmonic thermal behavior of the sample with cooling enabled excellent agreement between experiment and theory to be achieved, emphasizing the quality of the applied model, and the observed spectral peaks could be assigned to specific atomic motions in the solid. Form I and Form II polymorphs of ribavirin were both investigated with ss-DFT to understand the different aspects governing the relative stabilities of these solids. The ss-DFT simulations of the polymorph energies revealed that Form II is more stable at all temperatures due to a stronger cohesive energy than Form I; however, ribavirin in Form I has a significantly lower conformational energy. The finding of monotropism appears to conflict with the reported enantiotropism of the ribavirin polymorphs but ultimately confirms that crystal defects in the real samples greatly affect the thermodynamic relationship of the crystals.

## I Introduction

The polymorphic behavior of solid-state pharmaceutical molecules is of great importance in the formulation and application of medications.^1,2^ Polymorphism can be defined as when a crystalline solid has more than one three-dimensional arrangement of its components, with distinct differences often found in both molecular shape and intermolecular contacts. Polymorphs can have vastly different physical and chemical properties such as changes in aqueous solubility, which in the case of pharmaceuticals can modify the ability of a drug to perform its medical function.^3−5^ Due to the ramifications of polymorphism on drug efficacy, it is important to identify, differentiate, and study polymorphs to improve drug design and production.^6^

Vibrational spectroscopy techniques operating in the sub-200 cm^–1^ spectral region, including terahertz time-domain spectroscopy (THz-TDS) and low-frequency Raman spectroscopy (LFRS), are particularly useful for examining crystalline polymorphs.^7−10^ The vibrations present in this region involve large-amplitude motions of all components within the crystal and therefore offer new insights into internal torsional coordinates, external intermolecular motions, and the three-dimensional packing of the unit cell contents.^7,9,11^ Experimental low-frequency vibrational spectroscopy can be combined with solid-state density functional theory (ss-DFT) to further explore these spectra and the energetics involved in polymorphism.^12,13^ Given the unique material-specific character of sub-200 cm^–1^ vibrations, ss-DFT is valuable because it enables unambiguous spectral assignments and reveals the molecular origins of the measured spectra. In addition to assigning observed spectra to specific atomic-level motions, ss-DFT also allows for detailed investigations of the energetic factors involved in polymorph structures. This allows elucidation of the reasons underlying the differing stabilities of various solid forms.^12,14,15^

While THz-TDS and LFRS experimental results have been reported for numerous pharmaceutical compounds, very little low-frequency vibrational data has appeared for the important class of antiviral drugs based on nucleoside analogues.^16^ The THz spectrum of acyclovir was reported in 2015,^17^ and the THz spectra of ribavirin and entecavir appeared in 2022 (during the preparation of the current manuscript).^18^ While not noted in the publication, the observed ribavirin spectrum exhibited unusually narrow features at room temperature, but no temperature-dependent study was performed. The width of the spectral features in the low-frequency spectra of molecular solids can generally be explained by the anharmonic character of the vibrations within the solid, with lesser anharmonic character yielding narrower peak widths. The anharmonicity of lattice vibrations in molecular crystals has been found to be a significant factor for understanding polymorph stability and phase transitions, with several studies appearing that utilize THz-TDS and ss-DFT methods for evaluating this phenomenon.^19−21^ In the current study, we apply both THz-TDS and LFRS with cryogenic cooling and ss-DFT in the research of crystalline ribavirin to better understand its lattice vibrations and solid-state properties of this nucleoside analogue.

**Figure 1:**
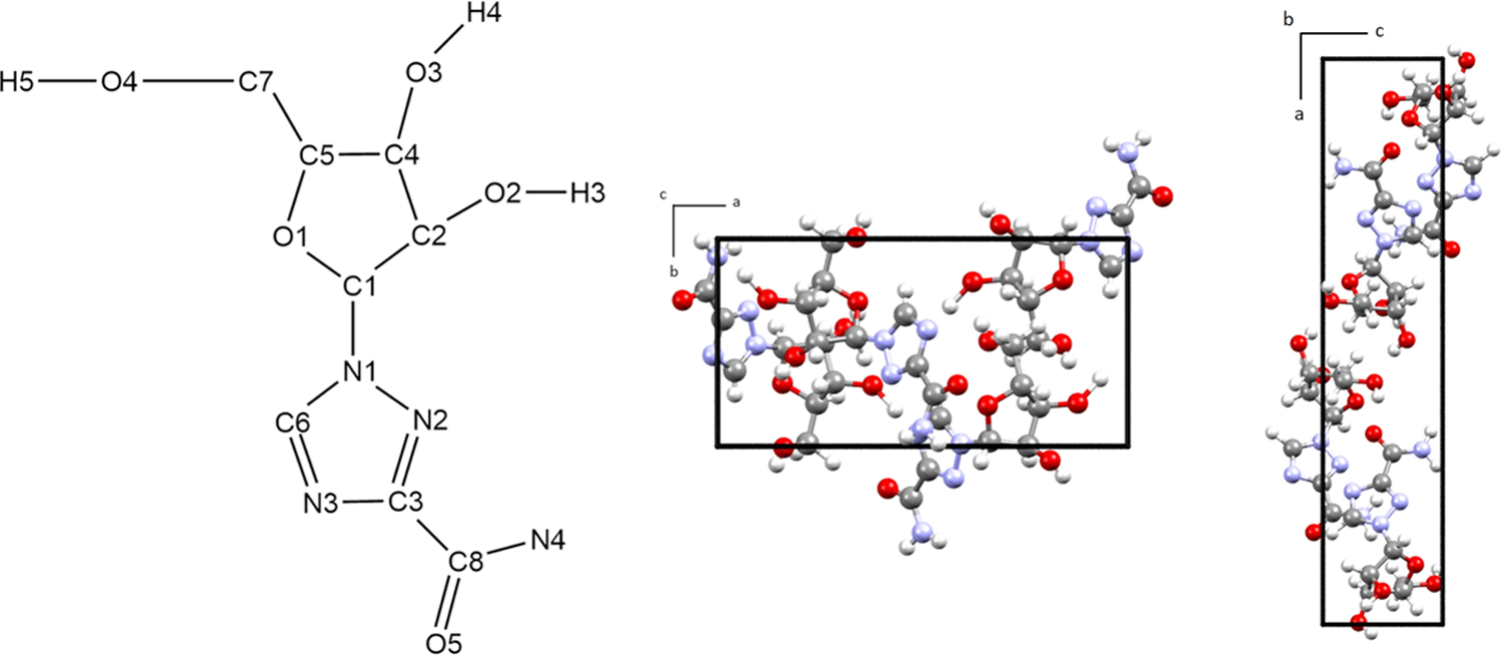
Two-dimensional representation of ribavirin with relevant hydrogens labeled (left), crystallographic unit cells for R-I (middle), and R-II (right).

The antiviral properties of ribavirin (C_8_H_12_N_4_O_5_) were discovered in 1972, and since then it has been primarily used to treat respiratory infections and used in combination with other medicines for hepatitis C.^22−24^ More recently, ribavirin has attracted interest as a possible therapeutic against SARS-CoV-2.^25^ In the solid state, it can be found in two crystalline polymorphs (known as Form I and Form II, Figure 1).^26^ Ribavirin Form II (R-II) is considered the thermodynamically stable polymorph and is found in commercial products.^22^ However, previous experiments have demonstrated that mechanical processing can cause crystal defect-induced conversion between the two enantiotropic polymorphs, which could present a risk during manufacturing and storage of ribavirin.^27^ The relationship between these two polymorphs is also interesting because the stability ranking violates the typical density rule.^3^ While this rule does not always hold, these polymorphs exhibit atypical behavior where R-II (density = 1.587 g/cm^3^) is more stable but less dense than ribavirin Form I (R-I, density = 1.653 g/cm^3^).^27^

One important factor that directly affects the crystalline polymorphism of drug molecules is conformational flexibility or the ability of the molecules to achieve multiple three-dimensional shapes. The conformational flexibility of pharmaceutical compounds has long been a subject of study and the basis for designing and understanding drug function, generally represented as the structure–activity relationship.^28,29^ The conformations of several antivirals have been studied in this context, including acyclovir,^30^ peramivir,^31^ and ritonavir,^32,33^ to name a few. The conformation of ribavirin has been the subject of computational conformational analyses before, using both semiempirical^34,35^ and density functional theory methods.^36^ The potential energy surface governing torsion of the O1–C1–N1–C6 dihedral angle (Figure 1) has been a focus, as this is the coordinate primarily responsible for the conformation of the molecule in the solid state and also for its successful drug binding capability.^34^ The shape of a molecule is also strongly affected by intermolecular forces as it interacts with its environment (solid or otherwise) and this is exemplified by the fact that the conformation of a bound drug in an active site is typically different than that in solution.^37^ Therefore, the conformational energy associated with the shape of ribavirin is of particular interest here as it can provide insights into the crystallization mechanism from solution, but also is important for understanding the drug action of the molecule.

In the current work, the more stable R-II polymorph is investigated using THz-TDS and LFRS, and then ss-DFT is applied in the analysis of both ribavirin solid forms. These vibrational spectra and simulations provide uniquely identifying spectral features for ribavirin polymorphs that may be used for analytical purposes and confirm the nearly harmonic vibrational properties of crystalline ribavirin. The observed vibrations represent intermolecular motions that yield insight into the crystal packing and intramolecular torsions that are linked to the three-dimensional shape of ribavirin. The exact natures of the peaks in the measured spectra have been determined with ss-DFT, enabling the identification of the key torsional vibrations and how conformational and cohesive energies combine to dictate polymorph stability. The high quality of the ss-DFT results also highlights the important role that crystal defects serve in establishing the thermodynamic relationships of real samples.

## II Methods

### II.I Sample Preparation

Ribavirin was purchased from TCI America and used without further purification. Room-temperature powder X-ray diffraction (PXRD) was done on the ribavirin sample with a Bruker D2 Phaser diffractometer (Cu Kα radiation, λ = 1.54060 Å, 5–70° with 0.5 s per step). PXRD data were compared to data from the Cambridge Structural Database (CSD)^38^ and the ribavirin sample was confirmed to be R-II (P2_1_2_1_2_1_, *Z* = 4).^26^ The PXRD comparison can be found in the Supporting Information. Numerous attempts to grow significant quantities of R-I were unsuccessful and some results are provided in the Supporting Information.

### II.II Terahertz Time-Domain Spectroscopy (THz-TDS)

R-II was first ground and mixed with polytetrafluoroethylene (PTFE) and then pressed into 13 mm diameter x 3.04 mm thick pellets (2.2% w/w). A blank reference pellet of pure PTFE was used to ratio out any matrix absorption by the PTFE in preparation of the final terahertz data shown here. Room-temperature (295 K) and 20 K (closed-cycle helium cryostat) THz-TDS data were collected using a Toptica Photonics TeraFlash spectrometer (Munich, Germany). The instrument is based on a λ = 1.5 μm femtosecond fiber laser used to illuminate photoconductive switch elements, with an InGaAs 25 μm strip-line photoconductive antenna for THz generation, and a 25 μm InGaAs dipole photoconductive antenna for THz detection. A time window of 28 ps after the THz pulse center was used for subsequent analysis to avoid interfering pulse reflections and the time-domain waveform data sets were zero-padded to 1201 points. The zero-padded sample and blank waveforms were then Fourier-transformed using the Blackman window prior to ratioing of the two. The final THz-TDS spectra had a spectral range of 5–150.0 cm^–1^ and a spectral resolution of 1.1 cm^–1^. The absorption spectra are expressed in units of molar extinction coefficient (M^–1^ cm^–1^) where concentration is based on the concentration of the crystallographic unit cells in the sample.

### II.III Low-Frequency Raman Spectroscopy (LFRS)

A Coherent (Ondax) THz-Raman system (Santa Clara, CA) was used to take both room-temperature (295 K) and 78 K (liquid nitrogen) data using a laser centered at 784.7 nm and an Andor Shamrock DR-750 spectrograph with an iDus 416 CCD detector. For all measurements, finely ground powder was placed in a cryostat-mounted cuvette system with glass windows that allowed the sample to be cooled via liquid nitrogen as needed. At both 295 and 78 K, 225 acquisitions were taken with 3 s exposure times. The Raman spectra had a spectral range of 10–300 cm^–1^ and a spectral resolution of 0.6 cm^–1^. Atmospheric interference from N_2_ and O_2_ rotational transitions was identified from a spectrum taken of only air and then subtracted from the final Raman data using the Spectragryph spectroscopy software (version 1.2.15).^39^

### II.IV Theoretical Methods

CRYSTAL17^40^ was used to complete quantum mechanical simulations of R-I and R-II. For all of the calculations, the def2-TZVP^41^ basis set was used along with the Perdew–Burke–Ernzerhof (PBE)^42^ density functional augmented with Grimme’s London dispersion correction (D3) utilizing the Becke–Johnson damping correction^43−45^ and three-body repulsion Axilrod–Teller–Muto repulsion contributions (program keyword “ABC”).^46−48^ A pruned integration grid of 99 radial points and 1454 angular points was used for all calculations. For both R-I and R-II, 125 *k*-points were used in the irreducible Brillouin zone (SHRINK 9 9). For optimizations, vibrational frequencies, and energy calculations, the overlap-based truncation criteria for the bielectronic integrals (Coulomb and exchange, TOLINTEG) were set to 10^–10^, 10^–10^, 10^–10^, 10^–10^, and 10^–20^.

The computational approach used in this study is identical to that used in an earlier work on similarly complex organic crystals including biotin, α-lactose monohydrate, and L-cystine.^49^ In such studies, the use of a large basis set (greater number of functions) to describe the atomic/molecular electron densities in the crystals produces higher-quality predicted spectra with improved correlations with experiment.^50^ Given the inherent trade-off between basis set quality and simulation run time, the def2-TZVP basis set was the largest basis set of practical use for simulations of crystalline ribavirin with generally available computer hardware.

The Cambridge Structural Database (CSD)^38^ was used to obtain the starting experimental structures for the optimizations, with reference codes VIRAZL for R-I and VIRAZL01 for R-II (both reported at room temperature).^26^ For optimizations, an energy convergence of Δ*E* < 10^–8^ hartree was used. Two types of optimizations were done for R-II, to better simulate both the cold and room-temperature structures and resulting vibrational spectra. Fixed-lattice simulations were done to better approximate the room-temperature samples and results are provided in the Supporting Information. These were performed by setting the lattice dimensions to the measured room-temperature XRD values and allowing only the atom positions to optimize within these constraints. Full optimizations were done by allowing the lattice dimensions and the atom positions to simultaneously optimize and being a 0 K simulation, are more representative of low-temperature structures and spectra.

Vibrational frequency analyses were done for both the fixed-lattice and fully optimized structures for R-II and for only the fully optimized structure of R-I. For the normal mode vibrational frequency and energy calculations, an energy convergence of Δ*E* < 10^–10^ hartree was used. For the frequency calculations, each atom was displaced twice along each Cartesian axis and the determination of the numerical derivatives of the Hessian matrix was done with the central difference formula. The IR and Raman intensities were both calculated using the coupled-perturbed Hartree–Fock/Kohn–Sham (CPHF) approach.^51−53^ For the Raman intensities, parameters for the experimental temperature and laser wavelength were accounted for. Simulated spectra were also convolved with Lorentzian line shapes with empirical full width at half-maximum (FWHM) values to facilitate comparison with the experiment.

Energy analyses were also done on both polymorphs. The total electronic energy of the crystallographic unit cell can be broken down into conformational and cohesive energies. The conformational energy was found by extracting an individual ribavirin molecule (the asymmetric unit of both polymorphs is one ribavirin molecule) from the ss-DFT optimized crystal and the energy of the fixed conformation was calculated. To determine the cohesive energy, the conformational energies of all of the individual molecules in the unit cell were subtracted from the total electronic energy. Finally, the vibrational calculations were used to construct Gibbs free energy vs temperature curves from 0 to 500 K for each polymorph. The reported melting points are 450.6 K for R-I and 441.3 K for R-II.^27^

Isolated-molecule DFT simulations were also performed on single ribavirin molecules using Gaussian 16W (Revision C.01).^54^ This software was used to scan the potential energy surface associated with the O1–C1–N1–C6 dihedral angle and perform gas-phase geometry optimizations. The Gaussian calculations were set up to be as close to the CRYSTAL17 parameters as possible, using the same density functional, noncovalent corrections, and basis set, but with the DFT integration grid increased to a larger grid than the software default (integral = grid = superfine).

## III Results and Discussion

### III.I Structural Differences and Optimization

The R-I and R-II crystals were ss-DFT optimized and subsequent frequency analyses (vide infra) indicated the structures corresponded to minima on their respective potential energy surfaces. The fully optimized ss-DFT structure of R-I (calculated density = 1.652 g/cm^3^) had an average unsigned overall percent error in the unit cell dimensions of 0.42% with the largest error being 0.69% on the *c* axis and a volume error of 0.01%. The root-mean-squared deviation (rmsd) in the internal structure also showed low errors with an rmsd for the bond lengths of 0.01 Å, the bond angles of 1.05°, and the torsions of 1.30°. The rmsd for the intermolecular heavy-atom hydrogen bond lengths of R-I was 0.06 Å. The fully optimized ss-DFT structure of R-II (calculated density = 1.607 g/cm^3^) had an average unsigned overall percent error in the unit cell dimensions of 0.71% with the largest error being 1.50% on the *a* axis and a volume error of −1.31%. The R-II structure had an rmsd of 0.01 Å for the bond lengths, 0.80° for the bond angles, and 1.41° for the torsions. The rmsd for the intermolecular heavy-atom hydrogen bond lengths of R-II was 0.05 Å. The unit cell dimensions and fractional coordinates for both fully optimized polymorphs are provided in the Supporting Information.

### III.II Experimental and Simulated Terahertz Spectra

**Figure 2:**
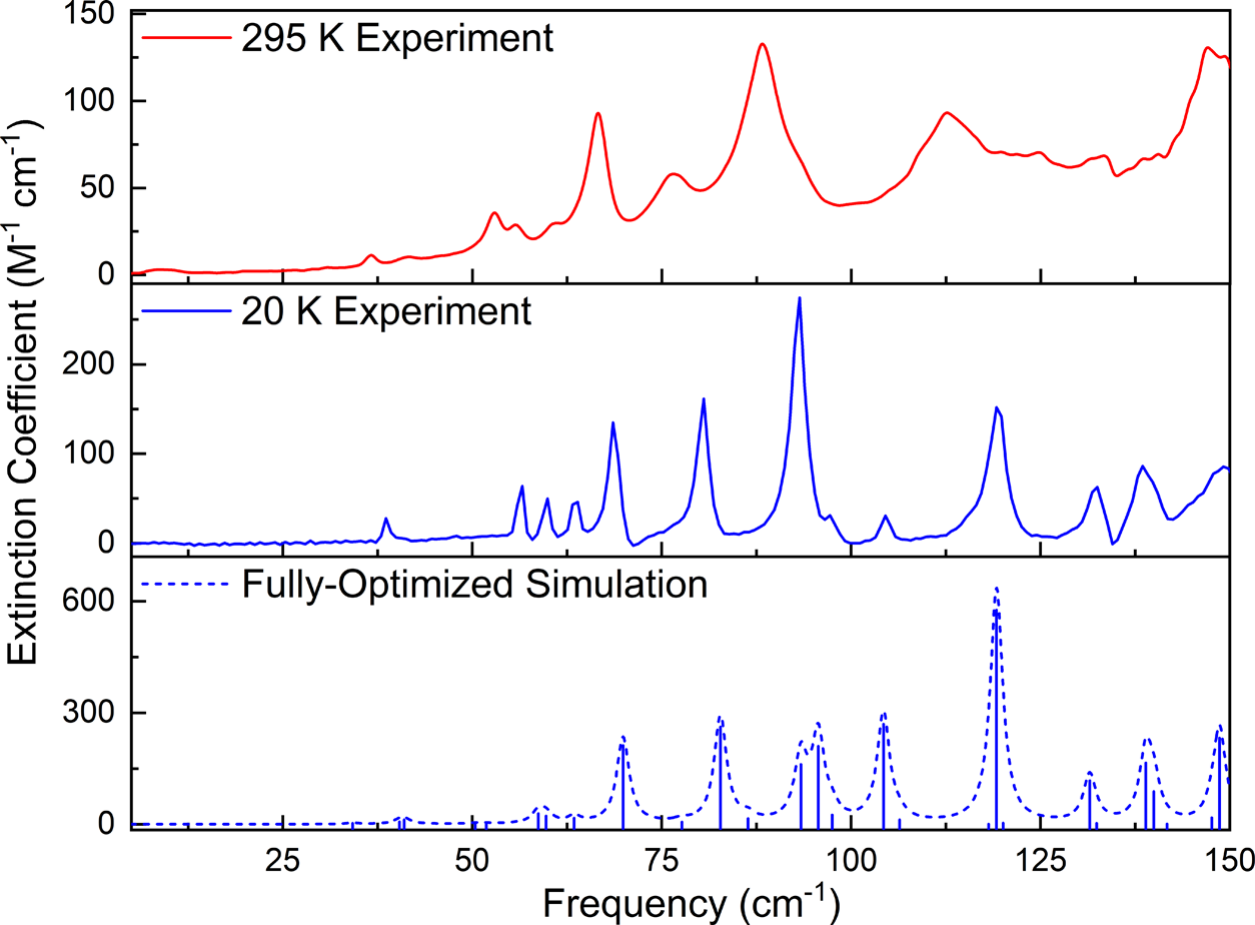
Experimental 295 K (red) and 20 K (blue) THz spectra for R-II from 5 to 150 cm^–1^. The simulated THz spectrum (dashed blue, 1.0 cm^–1^ FWHM) is based on the full-optimization calculations.

The experimental THz spectra of R-II recorded at ambient and low temperatures are shown in Figure 2. The spectra show distinct absorption features at both temperatures, but the features narrow and shift to higher energy with cooling as is typical.^55^ It is worth noting that in the case of R-II, the THz peaks are reasonably well resolved at 295 K (consistent with other reports)^18^ and there are only modest changes at 78 K. This is indicative of the R-II crystal lattice vibrations being more harmonic in character compared to other solids where much larger temperature effects have been observed that reveal their anharmonic natures.^56^ The apparent near-harmonic behavior of the R-II crystal is also consistent with the comparison of the full geometry optimization that deviated from the room-temperature unit cell volume by only 0.01%.

The experimental 20 K THz spectrum of R-II is shown in Figure 2 along with the fully optimized ss-DFT simulation which shows good agreement. A complete list of simulated IR-active vibrational modes for both the fixed-lattice and the fully optimized simulations can be found in the Supporting Information. Four major features can be identified in the 20 K THz-TDS spectrum at 69, 81, 93, and 119 cm^–1^. There are also a series of four smaller peaks between 35 and 65 cm^–1^ and two smaller peaks between 95 and 105 cm^–1^. The simulation reproduces the four most intense peaks well, with peaks at 69.9, 82.8, and 119.2 cm^–1^ corresponding to the experimental peaks at 69, 81, and 119 cm^–1^, respectively. The most intense feature at 93 cm^–1^ in the experimental spectrum originates from the two features in the simulation at 93.4 and 95.7 cm^–1^ that are split by a greater magnitude than what is measured. The average unsigned error between the 20 K and simulated THz spectral features is very low at 0.99 cm^–1^ and emphasizes the harmonic character of the R-II lattice vibrations. A list of 295 and 20 K THz-TDS peak positions with assigned simulated values is provided in the Supporting Information.

### III.III Experimental and Simulated Low-Frequency Raman Vibrational Spectra

**Figure 3:**
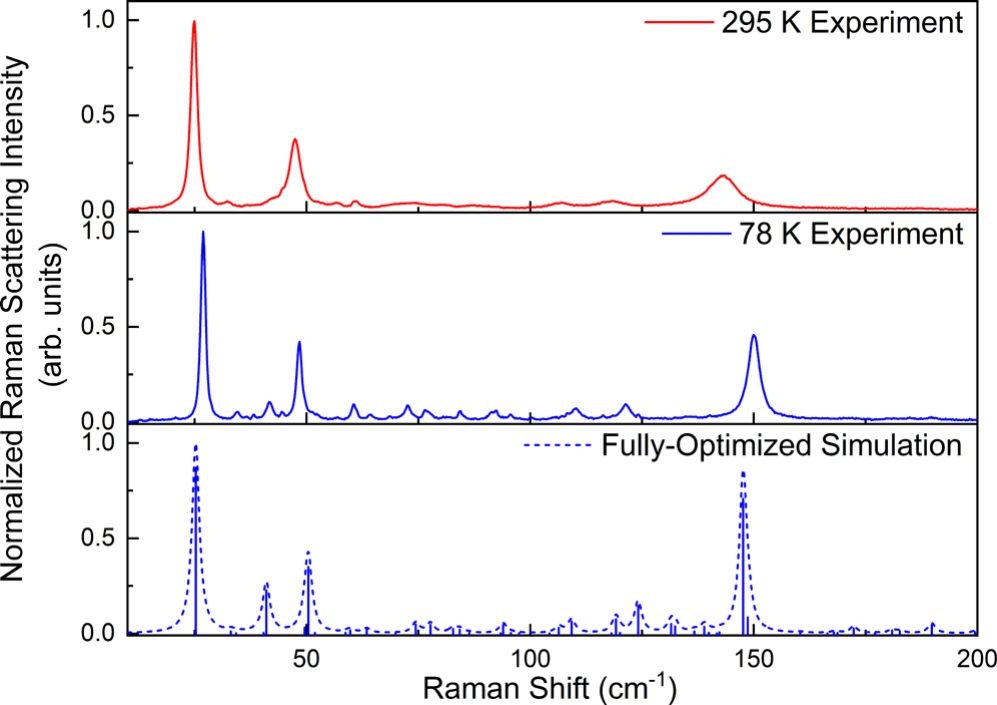
Experimental 295 K (red) and 78 K (blue) Raman spectra for R-II from 10 to 200 cm^–1^. Simulated Raman spectra (dashed blue, 1.0 cm^–1^ FWHM) are shown based on the full-optimization calculations.

The 295 and 78 K LFRS data for R-II are shown in Figure 3. The spectra both offer distinct spectral signatures for the R-II polymorph with several strong features being observed in each. As described for the THz-TDS data, the changes in the Raman spectrum with sample cooling are relatively small and support the harmonic nature assessment of the ribavirin crystal. Figure 3 also shows the fully optimized ss-DFT simulated Raman spectrum of R-II. As was seen with the THz-TDS data, the simulation does well at reproducing the lower temperature Raman spectrum. A complete list of simulated Raman-active vibrational modes for both the fixed-lattice and the fully optimized simulations can be found in the Supporting Information.

Within the 78 K Raman spectrum, three prominent peaks can be identified. The most intense peak is at 27 cm^–1^ in the experimental data and there are two other peaks at 48 and 150 cm^–1^ with several much weaker peaks between 50 and 145 cm^–1^. The simulation also shows three main peaks that are in good agreement with the experimental spectrum, at 25.2, 50.4, and 147.6 cm^–1^. The frequency positions of the predicted Raman peaks are very good, but the simulated intensities tend to be overestimated for some vibrations, including the simulated peaks at 41.0 and 147.6 cm^–1^, and the minor features above 150 cm^–1^. The average unsigned error between the 78 K and simulated Raman spectral peak positions is 1.51 cm^–1^, again a low value due to the minimal anharmonicity of R-II, but a slightly higher error than that found in the THz-TDS analysis. This may be a result of the different temperatures used in the LFRS and THz-TDS experiments since the lowest error between theory and experiment is expected to be achieved for the lowest sample temperature as anharmonicity is small but nonzero. A list of 295 and 78 K LFRS peak positions with assigned simulated values is provided in the Supporting Information.

### III.IV Types of Vibrations in the Sub-200 cm^–1^ Region

ss-DFT simulations also allow for the motion of the different vibrational modes to be determined and visualized. Lower-frequency modes generally represent vibrations with more collective motion that involve external coordinates (e.g., translations) while higher-frequency modes are more localized. This can be seen in the THz-TDS data with the simulated mode at 41.0 cm^–1^ in the simulation (corresponding to a 20 K experimental mode at 39 cm^–1^) and in the LFRS data at 25.2 cm^–1^ in the simulation (corresponding to 27 cm^–1^ in the 78 K spectrum) where both modes represent different rotational motions. At a higher frequency, modes such as that at 93.4 cm^–1^ in the THz-TDS simulation (93 cm^–1^ in the 20 K data) and 147.6 cm^–1^ in the LFRS simulation (150 cm^–1^ in the 78 K data) have motions that become more localized torsions in specific parts of the molecules.

### III.V Dihedral Angle Analysis

A critical dihedral angle in ribavirin is the O1–C1–N1–C6 coordinate (Figure 1) connecting the two rings that has been studied earlier for its relevance to drug binding.^34^ The experimental value for this dihedral angle in R-I is 10.406 and 119.019° in R-II as determined by single-crystal XRD. After the ss-DFT full optimizations, the dihedral angle was 9.992 and 120.509°, respectively, showing the ss-DFT results to be in excellent agreement with experiment. In terms of conformational energy, the conformation of ribavirin in the R-I polymorph is 15.95 kJ/mol lower in energy than in R-II, indicating the R-I conformation to be significantly more favorable. To further investigate this dihedral angle and the energy differences between the different conformations, single-molecule calculations were used to scan the dihedral angle potential energy surface and perform gas-phase geometry optimizations of the ribavirin molecules found in each solid-state polymorph.

First, a single ribavirin was extracted from both the R-I and R-II fully optimized crystals and all other dihedral angles and geometry parameters in the molecules were fixed at the ss-DFT values for both polymorphs. Then, rigid scans consisting of 36 steps in 10° increments were done of the O1–C1–N1–C6 dihedral in the R-I and R-II molecules to obtain the ring–ring potential energy surface for the ss-DFT optimized molecules. Once minima were found, finer dihedral scans with 1° increments were done near these positions.

In the R-I rigid dihedral scan, the starting dihedral angle of 9.992° was close to the lowest-energy dihedral angle found in the gas-phase potential energy surface scan at 17°. The energy difference between the R-I starting structure and the dihedral angle scan minimum was 0.34 kJ/mol. The R-II rigid dihedral scan produced a very different result as the starting dihedral angle was not near the minimum of the gas-phase potential energy surface. The lowest-energy dihedral angle found in the scan was 271° and was lower in energy than the starting angle by 9.74 kJ/mol (though still higher in energy than the scanned R-I by 6.48 kJ/mol). This indicates that the conformation of ribavirin in the R-II crystal is imposed by intermolecular forces and solid-state packing to a greater extent than in the R-I crystal. Plots of the energy versus dihedral angle are provided in the Supporting Information.

To further investigate the conformations of the two polymorphs, gas-phase full optimizations were done using the ss-DFT optimized structures as starting points. After gas-phase optimizations, the energy ranking was unchanged, but the R-I molecule was only 5.17 kJ/mol lower in energy than the R-II molecule. The dihedral angle changed significantly in both and became 26.607° in R-I and 11.588° in R-II. Normal mode analyses of both gas-phase optimized ribavirin structures showed them to be at potential energy surface minima. Complete optimizations of the R-I and R-II structures would ideally yield the same final geometry for the energetic minimum; however, the positioning of substituents on the ribosyl ring leads to differing local minima. The main difference in these two optimized structures is the position of the O4–H5 hydroxyl group attached to C7 (Figure 1). In R-I, the ss-DFT torsional angle of C5–C7–O4–H5 was 170.898° and in R-II it was -56.410. However, a lower-energy conformation can be achieved by two manual alterations of the optimized R-I molecule with subsequent geometry optimization in software. First, rotation of the O1–C1–N1–C6 dihedral angle to approximately -140° positions the hydroxymethyl group near the triazole ring to allow intramolecular hydrogen bonding from the hydroxyl to a ring nitrogen. Second, rotation of the N2–C3–C8–O5 dihedral angle from ∼0 to ∼180° moves the amide group to an energetically preferred orientation for isolated ribavirin. This amide orientation is the same as that of ribavirin in some co-crystalline solids^57^ and in crystals of 1H-1,2,4-triazole-3-carboxamide.^58^ Subsequent optimization of this manually prepared structure resulted in the lowest-energy conformation found in this study (25.20 kJ/mol lower than gas-phase fully optimized R-I), with an O1–C1–N1–C6 dihedral angle of −127.772°. This final structure (coordinates are provided in the Supporting Information) is lower in energy and yielded no negative vibrational modes, but it cannot be defined as the global minimum energy conformation for ribavirin as only a small portion of conformational space was considered. The structure is different than what has been previously reported and also similar in the sense that the lowest-energy conformers contained an intramolecular bond.^36^ It is expected that structures found through solid-state and gas-phase simulations (and experiments) will differ from solution-phase conformations in much the same way that drug structures differ in their solution and bound states.^37^

### III.VI Torsional Vibrations Involving the Ring–Ring Dihedral Angle

**Figure 4:**
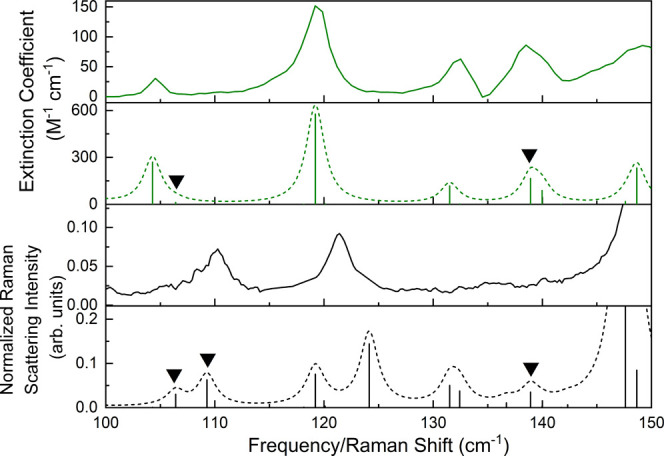
Experimental 20 K THz spectrum of R-II (green), the fully optimized ss-DFT simulated THz spectrum (1.0 cm^–1^ Lorentzian line shape) (dashed green), the experimental 78 K Raman spectrum of R-II (black), and the fully optimized ss-DFT simulated Raman spectrum (1.0 cm^–1^ Lorentzian line shape) (dashed black). Black triangles mark the torsional modes in each simulation.

The ss-DFT frequency analyses generate eigenvectors as part of the vibrational simulations and as mentioned earlier, this allows for the atomic motions associated with a vibration (Raman- or IR-active) to be investigated. Due to the importance of the O1–C1–N1–C6 dihedral angle, specific vibrations that involve this torsion can be identified, and the correlation of theory and experimental vibrational frequencies serves as a benchmark for the quality of the predicted ring–ring potential energy surface. Three ss-DFT calculated vibrations under 150 cm^–1^ that involve a large degree of motion in this dihedral can be found in each polymorph. These modes are 117.2, 123.1, and 127.5 cm^–1^ in R-I and 106.4, 109.3, and 138.9 cm^–1^ in R-II. Focusing on R-II for comparison to the experiments, the modes at 106.4 and 138.9 cm^–1^ are IR-active and all three modes are Raman-active. Based on the theoretical intensity values, the peak at 106.4 cm^–1^ should be very weak in the IR spectrum and in fact was not observed experimentally. The mode at 138.9 cm^–1^ is significantly stronger and can be seen in the experimental spectrum at approximately 138 cm^–1^, in very good agreement (Figure 4).

When considering the experimental Raman spectrum, the predicted vibrations at 106.4 and 109.3 cm^–1^ are represented by a somewhat broad asymmetric peak with a maximum at 110.3 cm^–1^ that is very close to the 109.3 cm^–1^ vibration, calculated to be the more intense of the pair (Figure 4). The predicted mode at 138.9 cm^–1^ cannot be unambiguously located in the experimental spectrum due to the weak Raman scattering intensity of the vibrations in this region. Despite the limited experimental opportunities for locating torsional vibrations of crystalline ribavirin, those that have been identified match well with the simulated frequencies and demonstrate that the ring–ring torsional potential energy is being well modeled.

### III.VII Analysis of Energy Factors in Crystallization

Due to the excellent accuracy of the structural and vibrational simulations compared to the experimental data, the ss-DFT simulations can be expected to provide meaningful analyses of the energetic factors driving the formation of the two polymorphs of ribavirin. For this purpose, only the fully optimized ss-DFT simulations are considered. After full optimizations, the total electronic energy of R-II is lower than R-I by 8.87 kJ/mol per asymmetric unit. These results are consistent with evidence indicating that R-II is thermodynamically stable under ambient conditions, while R-I is metastable and kinetically formed.^27^ The total electronic energy of both polymorphs can be further broken down into conformational and cohesive energies. These calculations show that R-II has a stronger cohesive energy of −24.82 kJ/mol per asymmetric unit, while R-I has a preferred conformational energy of −15.95 kJ/mol. The cohesion energy difference is likely the driving factor for why R-II is the more stable polymorph under ambient conditions. The finding that ribavirin molecules possess a lower conformational energy in metastable R-I is consistent with Ostwald’s step rule which suggests that the molecular conformations will more closely resemble those in solution in the polymorph formed first upon initial crystallization.^3^

**Figure 5:**
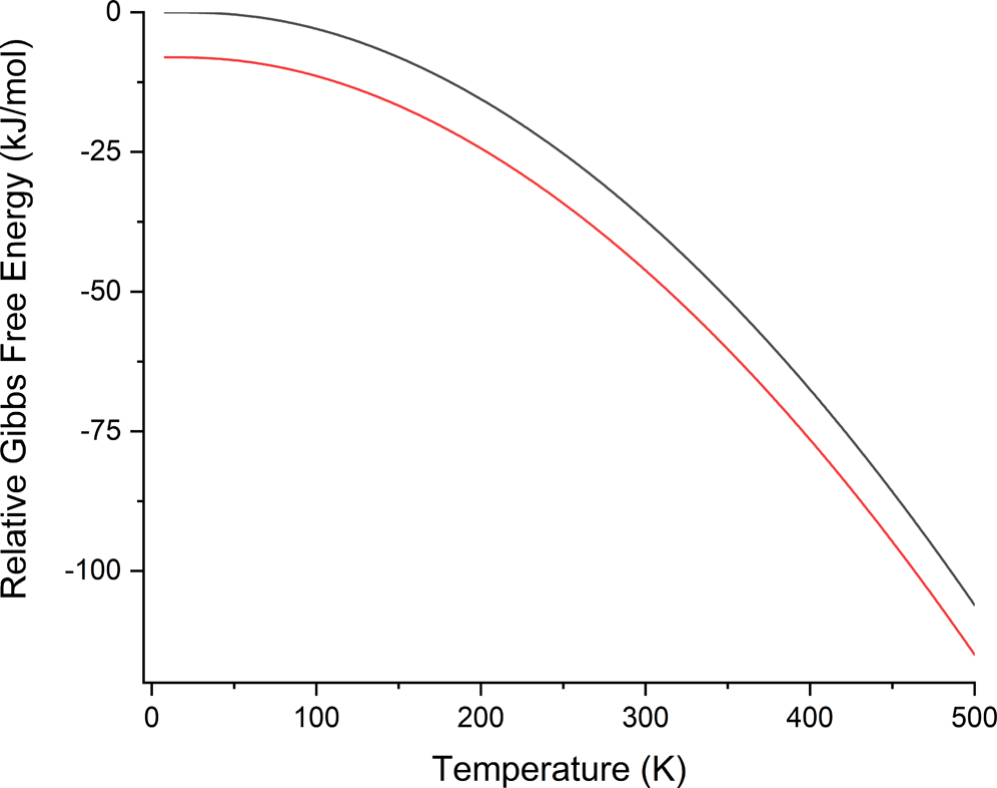
Relative Gibbs free energy curves for R-II (red) and R-I (black). All energies are per asymmetric unit (one ribavirin molecule). For clarity, the energy values have been set relative to a common zero.

In addition to examining ribavirin cohesion and conformation in the solid-state, ss-DFT can also be used to calculate Gibbs free energy curves of the polymorphs. The free energy calculations include the additional factors of vibrational energy and entropy to better understand the stability ranking of R-I and R-II. Figure 5 shows the Gibbs free energy versus temperature curves for R-I and R-II. These curves demonstrate that the two polymorphs have a monotropic relationship and that R-II is the thermodynamically stable form of crystalline ribavirin over the entire temperature range. With increasing temperature, the per molecule energy difference changes slightly with a separation of 8.05 kJ/mol at 20 K, 8.33 kJ/mol at 78 K, 8.96 kJ/mol at 295 K, and 8.93 kJ/mol at 446 K (melting point average of R-I and R-II). These results, along with the experimentally observed near-harmonic behavior of the R-II crystal, indicate that temperature alone is not the only driving force in previously reported ribavirin solid-state transformations.^27^

The determination of a monotropic relationship is seemingly in contrast with the findings of Vasa and Wildfong,^27^ which clearly established an enantiotropic relationship between R-I and R-II, with a transition temperature of 341.6 K. The ss-DFT simulated energies show no stability crossing, with R-II more stable at this temperature by 8.98 kJ/mol. Given the otherwise excellent correlation between theory and experiment, the discrepancy between the two results is almost certainly based on the important role that crystal defects have on the thermodynamic relationship of the ribavirin polymorphs, where the phase conversion is strongly facilitated by the defects. Since the simulations are based on perfect crystals, they do not account for the defects that can occur in natural crystals. Thus, the calculated monotropic phase relationship for the perfect crystals is consistent with experimental observations for pristine crystals of ribavirin (e.g., unmilled) and emphasizes that crystal defects caused by material processing during manufacturing enable enantiotropic behavior.

## IV Conclusions

Crystalline ribavirin exhibits strong and distinct low-frequency vibrational spectra that could be utilized for monitoring pharmaceutical manufacturing processes. Leveraging infrared and Raman spectroscopies of lattice vibrations in polymorphic solids with support from quantum mechanical simulations allows new insights to be achieved. The exploration of the low-frequency vibrational spectra of the R-II polymorph and the energetics involved in the stability of both R-I and R-II demonstrate the strengths of this combined approach. By investigating the conformation of the ribavirin molecules within the R-I and R-II crystals, it is apparent how the conformation of R-II in the solid state is enforced by intermolecular forces and that the conformation can change dramatically between solid-state and isolated molecules. Further energetic analysis shows that the perfect crystalline polymorphs are monotropic and R-II is more stable than R-I in terms of Gibbs free energy over a wide temperature range, driven primarily by its stronger cohesive energy. Overall, these findings confirm the metastability of the R-I polymorph and provide further evidence of a crystal defect-derived pathway that promotes the experimental enantiotropic growth of this crystal form of ribavirin.

An important discovery in this work is the relatively minor sensitivity of the low-frequency vibrational spectra of ribavirin to reduced temperatures, as this reflects the harmonic nature of the vibrations in this crystalline sample. Since the simulated vibrational spectra are based on a purely harmonic model, the correlation between experiment and theory will always be higher for samples that exhibit little vibrational anharmonicity and this is certainly true for ribavirin. Comparing the current results to the outcomes reported in earlier studies^49^ indicates that the enhanced agreement between theory and experiment for ribavirin is largely attributable to the harmonic character of the ribavirin crystal. These findings for ribavirin are similar to that for carvedilol (form II),^50^ where the experimental LFRS data shows very little change with cooling and the ss-DFT simulation of the sub-300 cm^–1^ vibrations provides excellent agreement with the observed peak positions. Ultimately, the harmonic character of the lattice vibrations in R-II may be directly related to the experimental difficulty in growing crystals of the R-I polymorph without the aid of crystal defects, as anharmonicity in this spectral region has been identified as serving an important role in such phase transitions.^20,21^

## Supporting Information Available

PXRD patterns for pure R-II sample and mixture of R-II and R-I samples with comparison to database patterns for R-I and R-II; FTIR spectrum of R-II from 80 to 300 cm^–1^; experimental 295 and 20 K THz spectra for R-II with simulated THz spectra based on fixed-lattice and full-optimization calculations; experimental 295 K and 78 K Raman spectra for R-II with simulated THz spectra based on fixed-lattice and full-optimization calculations; list of solid-state DFT IR- and Raman-active modes for R-I and R-II full optimization and R-II fixed-lattice optimization; energy versus dihedral angle curves for R-I and R-II; unit cell dimensions and fractional coordinates for the R-I and R-II full optimizations; atomic coordinates for R-I final conformation from isolated-molecule calculations; and lists of experimental THz and Raman peak positions (room temperature and cold) and assigned simulated frequencies for R-II (PDF)
