# Supplementary material for: Low-Frequency Vibrational Spectroscopy and Quantum Mechanical Simulations of the Crystalline Polymorphs of the Antiviral Drug Ribavirin
Source: Mol Pharm. 2022 Aug 11;19(9):3385–93. doi: 10.1021/acs.molpharmaceut.2c00509 (PMC9449968; doi:10.1021/acs.molpharmaceut.2c00509)
Supplement: Supplementary file 1 — mp2c00509_si_001.pdf [file mp2c00509_si_001.pdf]

Low-Frequency Vibrational Spectroscopy and Quantum Mechanical Simulations of the Crystalline Polymorphs of the Antiviral Drug Ribavirin

Margaret P. Davis and Timothy M. Korter*

Department of Chemistry, Syracuse University, 1-133 Center for Science and Technology, Syracuse, New York 13244-4100, United States

Supporting Information

Figure S-1. Room temperature PXRD patterns of R-II (black) and CSD published pattern[S-R1] (red).

Figure S-2. PXRD of a mixture of R-I and R-II (blue, top) with peaks attributed to R-I marked with red asterisks, pure R-I (red, middle), and pure R-II (black, bottom). [S-R1]

Figure S-3. FTIR spectrum of R-II from 80 to 300 cm^{-1}.

Table S-1. Solid-state DFT optimized lattice parameters for R-I and R-II.

Table S-2. Solid-state DFT optimized atomic positions in fractional coordinates for the asymmetric unit cell of R-I.

Table S-3. Solid-state DFT optimized atomic positions in fractional coordinates for the asymmetric unit cell of R-II.

Figure S-4. Experimental 295 K (solid red line) and 20 K (solid blue line) THz spectra for R-II from 5 to 120 cm^{-1}. Simulated THz spectra are shown based on fixed-lattice (dashed red line, 2.0 cm^{-1} FWHM) and full-optimization (dashed blue line, 1.0 cm^{-1} FWHM) calculations.

Figure S-5. Experimental 295 K (solid red line) and 78 K (solid blue line) Raman spectra for R-II from 10 to 200 cm^{-1}. Simulated Raman spectra are shown based on the fixed-lattice (dashed red line, 2.0 cm^{-1} FWHM) and full-optimization (dashed blue line, 1.0 cm^{-1} FWHM) calculations.

Table S-4. Solid-state DFT IR-active modes with frequencies (cm^{-1}), intensities (km/mol), and mode symmetries for R-I full optimization.

Table S-5. Solid-state DFT Raman-active modes with frequencies (cm^{-1}), relative intensities (normalized to 1000), and mode symmetries for R-I full optimization at 78 K.

Table S-6. Solid-state DFT IR-active modes with frequencies (cm^{-1}), intensities (km/mol), and mode symmetries for R-II full optimization.

Table S-7. Solid-state DFT Raman-active modes with frequencies (cm^{-1}), relative intensities (normalized to 1000), and mode symmetries for R-II full optimization at 78 K.

Table S-8. Solid-state DFT IR-active modes with frequencies (cm^{-1}), intensities (km/mol), and mode symmetries for R-II fixed-lattice optimization.

Table S-9. Solid-state DFT Raman-active modes with frequencies (cm^{-1}), relative intensities (normalized to 1000), and mode symmetries for R-II fixed-lattice optimization at 295 K.

Figure S-6. Relative energy versus dihedral angle (O1-C1-N1-C6) curves for R-I and R-II.

Table S-10. Coordinates (angstroms) for R-I final conformation from Gaussian simulations.

Table S-11. Experimental and simulated peak positions (cm^{-1}) in the terahertz spectrum of R-II.

Table S-12. Experimental and simulated peak positions (cm^{-1}) in the Raman spectrum of R-II.

References

S-R1. Prusiner, P.; Sundaralingam, M., The Crystal and Molecular Structures of Two Polymorphic Crystalline Forms of Virazole (1-B-D-Ribofuranosyl-1, 2, 4-Triazole-3-Carboxamide). A New Synthetic Broad Sprectrum Antiviral Agent. *Acta Crystallographica Section B: Structural Crystallography and Crystal Chemistry* **1976,** *32* (2), 419-426.

S-R2. Macrae, C. F.; Sovago, I.; Cottrell, S. J.; Galek, P. T.; McCabe, P.; Pidcock, E.; Platings, M.; Shields, G. P.; Stevens, J. S.; Towler, M., Mercury 4.0: From Visualization to Analysis, Design and Prediction. *Journal of Applied Crystallography* **2020,** *53* (1), 226-235.

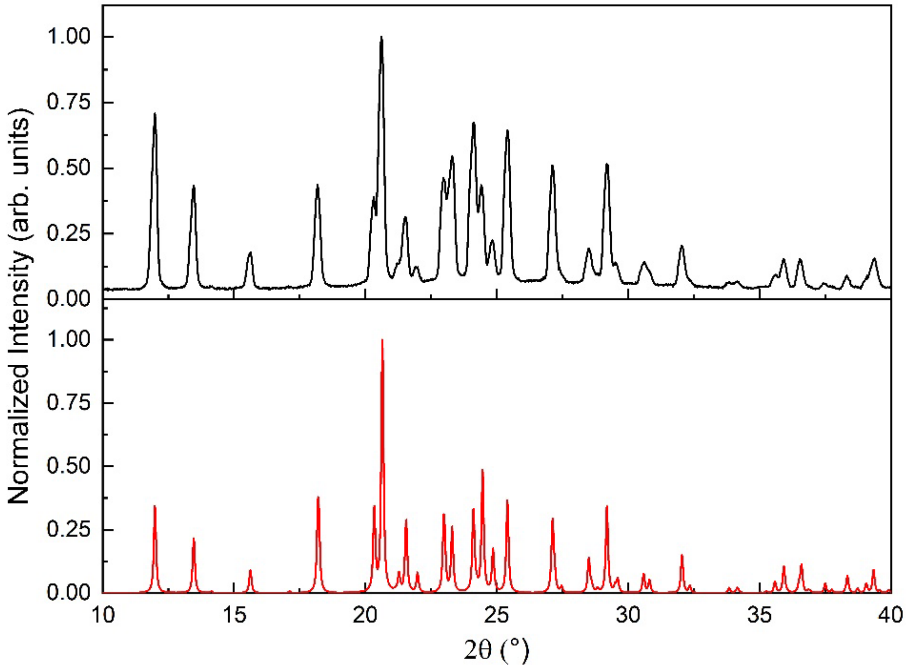

Figure S-1. Room temperature PXRD patterns of R-II (black) and CSD published pattern[S-R1] (red).

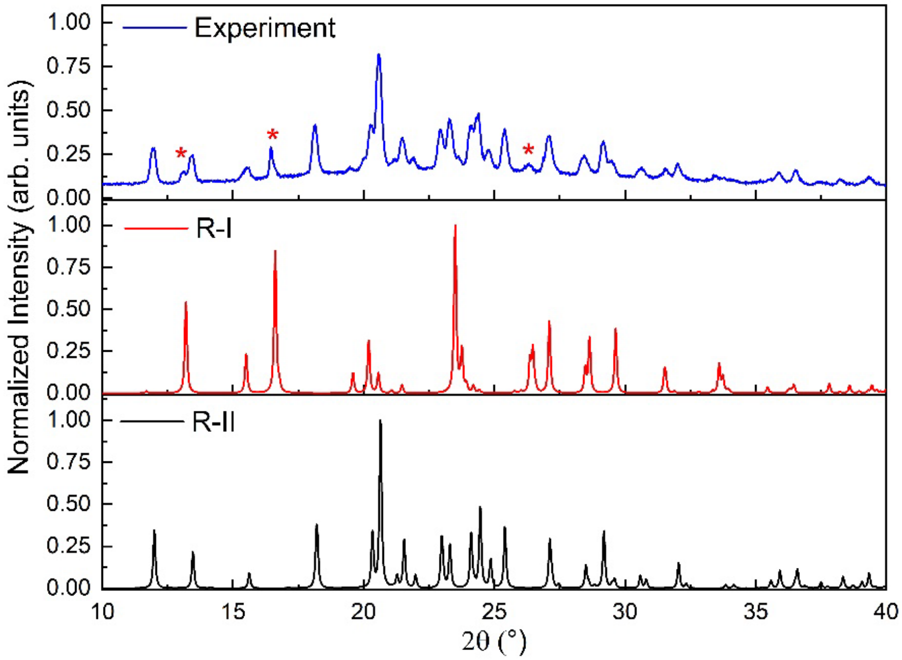

Figure S-2. PXRD of a mixture of R-I and R-II (blue, top) with peaks attributed to R-I marked with red asterisks, pure R-I (red, middle), and pure R-II (black, bottom). [S-R1]

Figure S-2. shows the PXRD pattern of the mixture along with the PXRD patterns of pure R-I and R-II predicted from CSD data[S-R1] and generated using Mercury[S-R2]. Peaks marked with red asterisks in the mixture PXRD pattern represent peaks attributed to R-I and account for about 10% of the sample. The mixture of R-I and R-II was formed from the melt of R-II after heating to 169-170 °C on a hot plate.

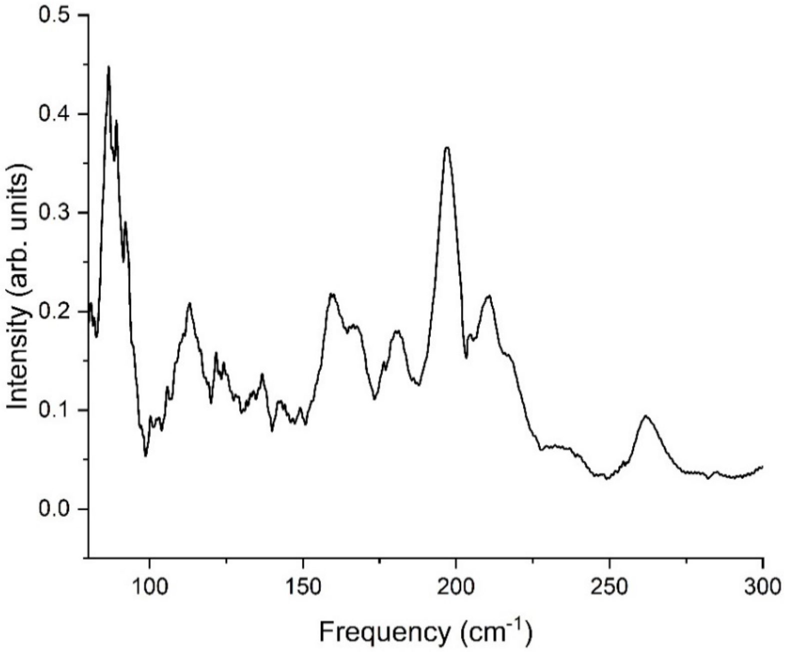

Figure S-3. FTIR spectrum of R-II from 80 to 300 cm^{-1}.

A Bruker Invenio FTIR with a wide-range beamsplitter (T240-T) was used to confirm peaks at the end of the usable range for the Toptica Photonics TeraFlash. This data was taken by Dr. Kateryna Kushnir in Prof. Michael Ruggiero's group at the University of Vermont. The Bruker Invenio FTIR has a usable spectral range from 80 to 6,000 cm^{-1} with a spectral resolution of 0.5 cm^{-1}. The experiment was done at room temperature (290 K) and the sample was ground first then placed on a diamond ATR crystal and the entire system was purged with nitrogen gas during the experiment.

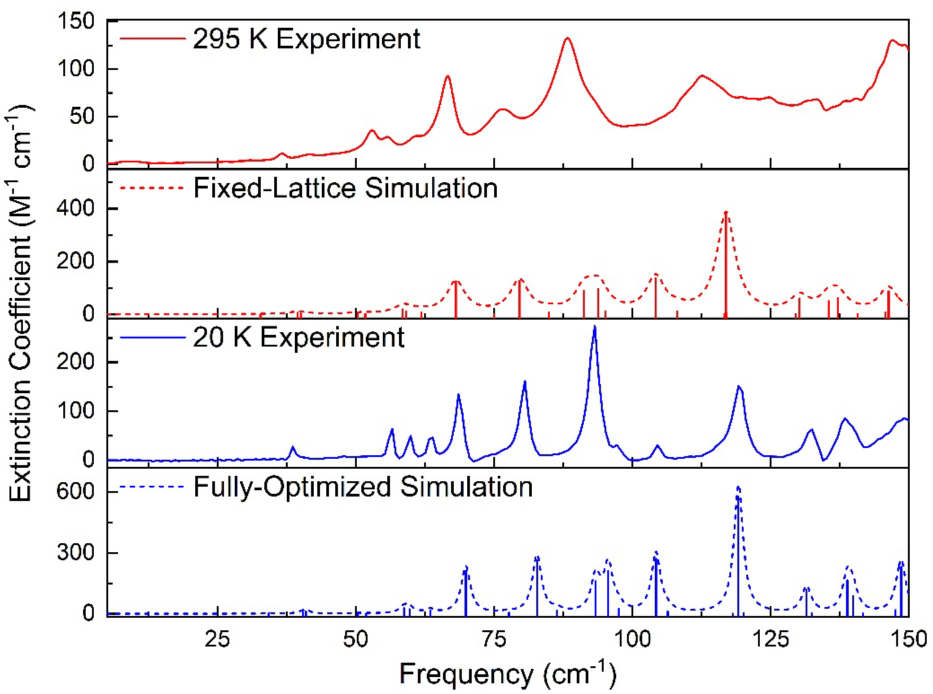

Figure S-4. Experimental 295 K (solid red line) and 20 K (solid blue line) THz spectra for R-II from 5 to 150 cm^{-1}. Simulated THz spectra are shown based on fixed-lattice (dashed red line, 2.0 cm^{-1} FWHM) and full-optimization (dashed blue line, 1.0 cm^{-1} FWHM) calculations.

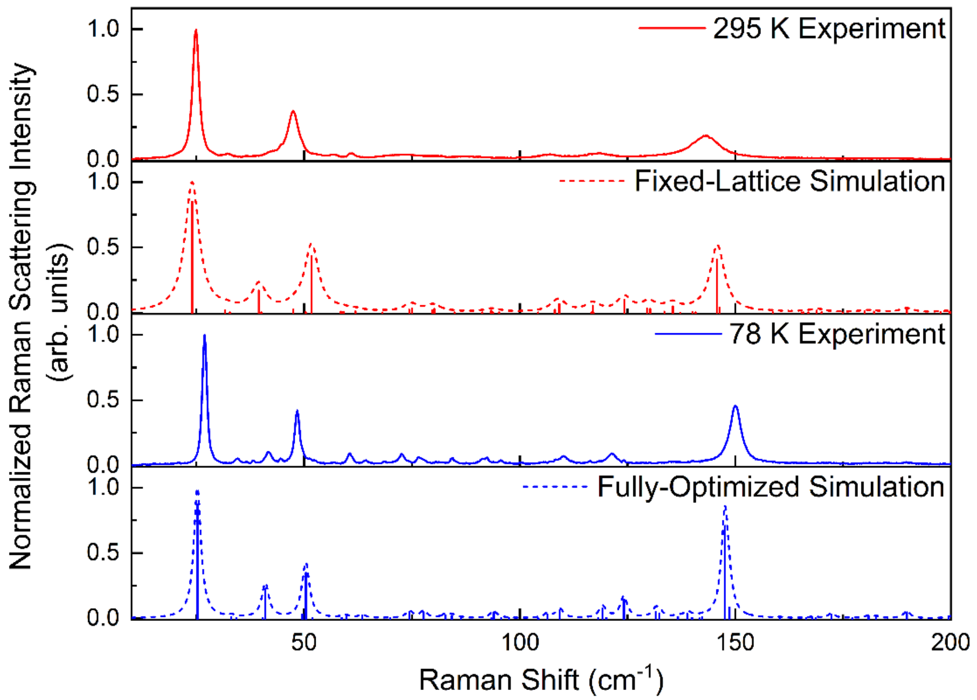

Figure S-5. Experimental 295 K (solid red line) and 78 K (solid blue line) Raman spectra for R-II from 10 to 200 cm^{-1}. Simulated Raman spectra are shown based on the fixed-lattice (dashed red line, 2.0 cm^{-1} FWHM) and full-optimization (dashed blue line, 1.0 cm^{-1} FWHM) calculations.

Table S-1. Solid-state DFT optimized lattice parameters for R-I and R-II.

		Lattice Parameters		
	Space Group	*a* (Å)	*b* (Å)	*c* (Å)
R-I	$P2_12_12_1$	14.7681	7.5129	8.8442
R-II	$P2_12_12_1$	24.6854	7.7504	5.2778

Table S-2. Solid-state DFT optimized atomic positions in fractional coordinates for the asymmetric unit cell of R-I.

Atom Type	X/A	Y/B	Z/C
C	-3.37638E-01	-2.82838E-02	2.15836E-01
C	-2.41508E-01	-2.77943E-03	2.77522E-01
C	-4.89746E-01	1.14142E-01	4.97970E-01
C	-2.14927E-01	-1.96392E-01	3.17559E-01
C	-2.61179E-01	-3.00830E-01	1.89714E-01
C	-4.60246E-01	-1.35767E-01	3.98239E-01
C	-2.86382E-01	-4.92464E-01	2.18267E-01
C	4.67268E-01	2.50231E-01	-4.03402E-01
H	-3.53410E-01	7.24155E-02	1.29309E-01
H	-2.40658E-01	8.38968E-02	3.77924E-01
H	-1.44979E-01	1.53373E-01	2.08163E-01
H	-9.74606E-02	-3.16007E-01	3.80503E-01
H	-3.66395E-01	3.64944E-01	3.48936E-01
H	-2.45634E-01	-2.31191E-01	4.27394E-01
H	-2.16360E-01	-2.98149E-01	8.99182E-02
H	-4.55456E-01	-2.74965E-01	3.66203E-01
H	-3.18865E-01	4.54892E-01	1.15177E-01
H	-2.23015E-01	4.33521E-01	2.37691E-01
H	-4.52868E-01	4.51609E-01	-4.87712E-01
H	4.61687E-01	-4.81011E-01	-3.61866E-01
N	-4.07412E-01	-9.93696E-03	3.34775E-01
N	-4.25437E-01	1.50353E-01	3.96289E-01
N	4.86348E-01	-6.08528E-02	-4.98114E-01
N	4.93377E-01	4.19249E-01	-4.21521E-01
O	-3.43021E-01	-1.99953E-01	1.54970E-01
O	-1.88019E-01	6.71008E-02	1.59696E-01
O	-1.19568E-01	-2.12588E-01	3.20372E-01
O	-3.46465E-01	4.90679E-01	3.44836E-01
O	4.08592E-01	2.00566E-01	-3.11050E-01

Table S-3. Solid-state DFT optimized atomic positions in fractional coordinates for the asymmetric unit cell of R-II.

Atom Type	X/A	Y/B	Z/C
C	3.68142E-01	4.89043E-01	4.17015E-01
C	4.11659E-01	-4.67838E-01	-3.87150E-01
C	2.47960E-01	2.56693E-01	-4.88798E-01
C	4.45926E-01	3.64465E-01	-3.91513E-01
C	4.40519E-01	3.01482E-01	3.32138E-01
C	3.11417E-01	3.29345E-01	-2.40975E-01
C	4.31032E-01	1.08819E-01	3.17833E-01
C	1.95439E-01	1.85758E-01	4.20940E-01
H	3.50679E-01	-3.95150E-01	3.29848E-01
H	3.95628E-01	-4.42257E-01	-1.96157E-01
H	4.63000E-01	-2.69772E-01	-3.53712E-01
H	-4.77626E-01	4.55560E-01	-4.22327E-01
H	3.94414E-01	-2.89384E-02	5.24746E-02
H	4.26952E-01	2.70158E-01	-2.62761E-01
H	4.76418E-01	3.36599E-01	2.19621E-01
H	3.37650E-01	3.47627E-01	-7.74147E-02
H	4.64888E-01	4.27420E-02	4.14218E-01
H	3.93464E-01	7.76893E-02	4.21465E-01
H	2.20130E-01	1.96419E-01	4.04321E-02
H	1.52678E-01	1.35166E-01	1.00318E-01
N	3.22499E-01	3.87304E-01	-4.78446E-01
N	2.82203E-01	3.41967E-01	3.61444E-01
N	2.64241E-01	2.46715E-01	-2.41824E-01
N	1.89485E-01	1.70279E-01	1.70502E-01
O	3.94664E-01	3.94447E-01	2.24944E-01
O	4.39713E-01	-3.23676E-01	-4.85826E-01
O	4.99539E-01	3.84144E-01	-3.03489E-01
O	4.26116E-01	5.22633E-02	6.11114E-02
O	1.59727E-01	1.49350E-01	-4.18909E-01

Table S-4. Solid-state DFT IR-active modes with frequencies (cm^{-1}), intensities (km/mol), and mode symmetries for R-I full optimization.

Mode Label (ν)	Frequency (cm^{-1})	Intensity (km/mol)	Mode Symmetry	Mode Label (ν)	Frequency (cm^{-1})	Intensity (km/mol)	Mode Symmetry
5	39.19	0.13	B1	54	214.08	25.56	B2
6	43.67	0.16	B2	55	240.53	8.54	B3
8	57.28	0.00	B3	56	242.93	2.98	B1
9	64.66	0.04	B2	58	245.14	24.30	B1
10	72.23	0.03	B3	59	245.36	1.16	B2
11	72.71	4.88	B1	60	246.49	1.30	B3
15	82.38	12.43	B1	61	256.92	4.83	B3
16	83.90	20.91	B2	62	258.63	0.15	B1
17	85.95	1.67	B3	63	268.72	46.97	B2
18	86.44	1.16	B3	65	278.79	50.69	B2
19	86.98	54.49	B1	67	281.04	40.45	B3
21	90.10	32.10	B2	69	282.33	22.57	B1
22	94.08	0.01	B2	70	283.91	12.80	B2
23	102.21	0.00	B1	71	296.67	0.11	B1
25	105.35	0.16	B3	72	296.67	17.19	B3
26	110.14	21.23	B2	73	319.06	9.09	B3
27	110.86	18.94	B1	74	319.48	83.37	B1
28	110.92	4.26	B3	75	325.62	7.72	B1
29	113.54	5.36	B1	77	329.94	50.03	B2
30	114.27	15.47	B3	78	330.10	9.21	B3
31	117.24	9.69	B2	80	362.51	125.06	B2
33	123.10	0.00	B3	82	381.12	27.41	B2
34	123.14	4.18	B2	83	384.88	44.14	B1
36	127.50	3.94	B1	84	386.12	225.85	B3
37	130.21	40.03	B1	86	414.05	11.41	B2
38	133.20	23.82	B2	87	422.10	91.62	B1
40	140.33	8.18	B3	88	423.67	24.55	B3
42	145.92	9.94	B3	89	446.42	39.56	B2
43	147.39	0.08	B1	90	448.63	3.08	B3
44	158.07	7.23	B2	92	450.73	54.61	B1
45	168.33	26.17	B2	94	463.44	86.87	B3
46	171.80	1.31	B3	95	465.11	435.75	B1
48	184.56	1.02	B1	96	466.03	83.58	B2
49	187.44	7.33	B1	98	546.78	10.30	B2
51	191.86	3.19	B3	99	551.78	2.03	B1
52	195.10	17.08	B2	100	554.59	8.39	B3

101	563.19	9.65	B2	156	848.12	132.27	B3
102	563.83	3.24	B1	158	857.92	16.80	B1
103	564.23	50.71	B3	159	858.43	57.29	B2
106	614.52	47.92	B3	160	858.54	66.97	B3
107	619.85	484.19	B1	161	871.87	92.45	B2
109	623.96	31.83	B2	162	873.50	48.83	B3
110	624.95	91.62	B3	163	874.60	6.25	B1
111	627.24	194.04	B2	166	922.99	18.79	B1
112	629.46	15.99	B1	167	924.00	12.91	B3
113	641.76	288.20	B2	168	924.03	5.66	B2
114	642.69	201.33	B1	169	972.49	174.26	B3
115	647.13	24.92	B3	170	972.96	0.83	B2
118	667.99	94.77	B3	171	975.18	88.28	B1
119	671.88	414.74	B2	173	1012.87	1.68	B3
120	675.68	525.27	B1	174	1013.73	91.37	B2
121	685.11	79.96	B3	177	1015.16	41.33	B1
122	688.95	14.34	B1	178	1015.99	100.78	B2
123	691.94	13.54	B2	179	1016.36	4.14	B3
125	705.71	188.68	B2	180	1016.95	17.85	B1
127	708.78	83.97	B3	182	1058.55	0.14	B1
128	708.89	8.72	B1	183	1058.96	459.02	B3
130	714.49	5.98	B2	185	1060.51	115.42	B2
131	716.17	106.30	B1	186	1061.46	291.22	B2
132	720.72	1.98	B3	187	1061.61	207.15	B3
133	741.43	547.76	B1	188	1061.64	48.91	B1
134	743.37	15.88	B3	190	1073.47	1.13	B2
135	743.66	94.98	B2	191	1073.85	39.18	B3
137	745.90	3.56	B1	192	1075.29	182.94	B1
138	747.75	0.06	B2	193	1079.26	208.98	B1
140	761.25	9.27	B3	195	1082.15	1138.29	B2
141	783.44	0.05	B3	196	1092.56	85.27	B3
143	783.73	34.83	B1	197	1094.90	94.63	B3
144	784.36	0.45	B2	199	1096.22	59.48	B1
145	800.06	185.59	B1	200	1097.18	45.04	B2
147	801.26	191.96	B2	201	1104.54	236.95	B2
148	802.52	12.24	B3	203	1108.00	21.75	B1
149	840.70	112.43	B3	204	1108.41	161.60	B3
151	841.40	52.65	B2	206	1123.09	41.49	B1
152	841.82	0.00	B1	207	1126.54	2.78	B3
153	843.80	279.29	B2	208	1128.27	12.72	B2
155	846.16	15.09	B1	210	1160.21	10.45	B3

211	1160.56	242.20	B2	266	1399.88	19.28	B3
212	1160.85	145.36	B1	267	1400.80	84.47	B2
213	1197.30	109.76	B2	268	1401.13	2.55	B1
214	1197.94	0.33	B3	269	1420.67	96.83	B2
216	1200.51	3.42	B1	270	1421.32	59.11	B1
217	1221.12	35.41	B2	272	1421.58	30.67	B3
219	1223.26	0.01	B1	273	1438.32	75.34	B3
220	1225.79	83.01	B3	274	1438.73	7.54	B2
221	1228.42	17.82	B3	276	1442.40	27.07	B1
223	1231.35	1.71	B1	277	1457.94	226.97	B2
224	1236.49	69.78	B1	278	1460.83	43.66	B3
225	1236.92	33.18	B2	280	1463.81	444.02	B1
226	1238.71	0.01	B3	281	1464.28	0.53	B3
228	1239.52	20.00	B2	282	1465.66	74.06	B2
230	1253.13	4.39	B1	283	1465.76	51.31	B1
231	1254.50	4.66	B3	285	1473.61	78.19	B2
232	1255.22	53.39	B2	286	1473.74	38.40	B3
233	1272.24	104.16	B3	288	1476.57	1.96	B1
234	1272.79	93.45	B2	289	1490.07	338.83	B3
235	1276.59	0.78	B1	291	1493.36	14.41	B1
237	1285.82	14.91	B2	292	1493.70	6.71	B2
238	1287.89	21.91	B3	293	1596.11	305.49	B2
240	1292.82	95.81	B1	294	1597.85	253.30	B1
242	1302.68	481.70	B3	296	1604.28	157.67	B3
243	1303.83	22.37	B2	298	1656.68	942.74	B3
244	1305.51	0.69	B1	299	1681.68	1322.72	B2
245	1311.36	201.32	B3	300	1687.70	455.95	B1
247	1313.04	0.76	B2	302	2950.04	148.25	B1
248	1313.18	1.19	B1	303	2950.70	0.01	B3
249	1313.78	182.51	B1	304	2950.77	0.92	B2
250	1317.52	288.46	B2	305	2966.32	1.25	B3
252	1319.30	68.85	B3	306	2966.33	2.04	B2
254	1324.49	257.10	B2	307	2966.55	14.21	B1
255	1325.19	21.89	B3	310	2979.50	31.27	B1
256	1330.70	3.35	B1	311	2980.47	121.59	B2
258	1355.10	1.34	B3	312	2980.69	23.86	B3
259	1355.17	0.16	B1	314	2992.81	33.16	B1
260	1355.50	33.47	B2	315	2993.23	118.25	B2
262	1358.08	55.59	B1	316	2993.52	94.94	B3
263	1358.17	59.27	B3	317	2995.76	115.09	B2
264	1359.50	17.87	B2	318	2996.02	1.71	B3

319	2996.54	1.38	B1
321	3008.89	11.36	B1
323	3009.31	33.57	B2
324	3009.37	82.32	B3
325	3071.00	4756.28	B2
327	3080.59	3264.23	B3
328	3081.71	975.21	B1
329	3186.74	515.29	B3
331	3186.88	0.03	B1
332	3186.90	0.05	B2
333	3226.52	2321.33	B2
335	3230.52	5883.59	B3
336	3232.11	877.87	B1
338	3285.87	361.58	B3
339	3288.83	3336.36	B2
340	3295.96	1399.93	B1
341	3331.17	5069.87	B3
343	3331.62	276.21	B2
344	3333.72	61.04	B1
345	3530.29	1135.14	B2
347	3531.63	66.92	B3
348	3533.68	470.05	B1

Table S-5. Solid-state DFT Raman-active modes with frequencies (cm^{-1}), relative intensities (normalized to 1000), and mode symmetries for R-I full optimization at 78 K.

Mode Label (ν)	Frequency (cm^{-1})	Relative Intensity	Mode Symmetry	Mode Label (ν)	Frequency (cm^{-1})	Relative Intensity	Mode Symmetry
4	37.24	9.49	A	40	140.33	18.01	B3
5	39.19	0.15	B1	41	144.22	113.17	A
6	43.67	43.73	B2	42	145.92	20.57	B3
7	54.80	16.34	A	43	147.39	11.61	B1
8	57.28	3.56	B3	44	158.07	7.20	B2
9	64.66	6.30	B2	45	168.33	20.38	B2
10	72.23	31.94	B3	46	171.80	27.79	B3
11	72.71	43.72	B1	47	177.36	12.05	A
12	75.39	53.54	A	48	184.56	19.40	B1
13	79.62	16.84	A	49	187.44	26.98	B1
14	81.62	213.21	A	50	189.42	39.88	A
15	82.38	0.02	B1	51	191.86	14.03	B3
16	83.90	0.79	B2	52	195.10	0.35	B2
17	85.95	39.46	B3	53	213.97	8.49	A
18	86.44	18.27	B3	54	214.08	28.41	B2
19	86.98	27.71	B1	55	240.53	8.17	B3
20	88.17	49.22	A	56	242.93	5.94	B1
21	90.10	65.05	B2	57	244.95	7.14	A
22	94.08	10.33	B2	58	245.14	10.13	B1
23	102.21	1.22	B1	59	245.36	6.42	B2
24	105.23	40.84	A	60	246.49	0.12	B3
25	105.35	14.42	B3	61	256.92	5.88	B3
26	110.14	5.71	B2	62	258.63	24.02	B1
27	110.86	9.10	B1	63	268.72	1.43	B2
28	110.92	1.92	B3	64	269.54	23.42	A
29	113.54	26.61	B1	65	278.79	0.00	B2
30	114.27	0.02	B3	66	278.83	44.29	A
31	117.24	113.94	B2	67	281.04	6.96	B3
32	117.93	28.69	A	68	282.14	48.02	A
33	123.10	8.22	B3	69	282.33	0.02	B1
34	123.14	105.40	B2	70	283.91	11.72	B2
35	126.87	36.67	A	71	296.67	21.75	B1
36	127.50	105.85	B1	72	296.67	0.04	B3
37	130.21	101.81	B1	73	319.06	8.98	B3
38	133.20	247.02	B2	74	319.48	19.31	B1
39	136.88	35.88	A	75	325.62	0.00	B1

76	326.61	24.72	A	117	665.28	4.04	A
77	329.94	0.86	B2	118	667.99	0.91	B3
78	330.10	4.08	B3	119	671.88	0.67	B2
79	360.13	28.53	A	120	675.68	2.75	B1
80	362.51	2.56	B2	121	685.11	3.45	B3
81	380.10	163.07	A	122	688.95	32.57	B1
82	381.12	6.27	B2	123	691.94	3.41	B2
83	384.88	0.03	B1	124	699.25	26.70	A
84	386.12	0.26	B3	125	705.71	0.02	B2
85	413.73	4.89	A	126	707.74	81.90	A
86	414.05	24.30	B2	127	708.78	4.03	B3
87	422.10	23.84	B1	128	708.89	5.57	B1
88	423.67	0.13	B3	129	711.00	40.02	A
89	446.42	0.02	B2	130	714.49	1.60	B2
90	448.63	0.21	B3	131	716.17	13.57	B1
91	449.57	31.61	A	132	720.72	11.47	B3
92	450.73	41.19	B1	133	741.43	1.77	B1
93	462.30	15.01	A	134	743.37	1.82	B3
94	463.44	0.56	B3	135	743.66	9.55	B2
95	465.11	4.19	B1	136	744.17	14.09	A
96	466.03	11.50	B2	137	745.90	0.94	B1
97	544.00	29.32	A	138	747.75	23.62	B2
98	546.78	10.74	B2	139	753.85	6.36	A
99	551.78	3.39	B1	140	761.25	4.13	B3
100	554.59	24.45	B3	141	783.44	1.48	B3
101	563.19	2.74	B2	142	783.58	21.68	A
102	563.83	9.29	B1	143	783.73	3.97	B1
103	564.23	7.86	B3	144	784.36	3.13	B2
104	567.01	28.76	A	145	800.06	19.97	B1
105	614.36	16.86	A	146	800.15	24.23	A
106	614.52	0.82	B3	147	801.26	0.02	B2
107	619.85	3.43	B1	148	802.52	7.75	B3
108	621.34	35.09	A	149	840.70	0.37	B3
109	623.96	6.92	B2	150	841.39	72.59	A
110	624.95	24.57	B3	151	841.40	4.26	B2
111	627.24	0.22	B2	152	841.82	3.07	B1
112	629.46	12.09	B1	153	843.80	14.31	B2
113	641.76	0.43	B2	154	844.15	78.95	A
114	642.69	2.85	B1	155	846.16	5.24	B1
115	647.13	5.21	B3	156	848.12	6.28	B3
116	647.60	4.90	A	157	857.23	44.76	A

158	857.92	0.06	B1	199	1096.22	0.01	B1
159	858.43	12.67	B2	200	1097.18	3.59	B2
160	858.54	0.01	B3	201	1104.54	4.87	B2
161	871.87	16.16	B2	202	1106.76	15.80	A
162	873.50	0.16	B3	203	1108.00	0.00	B1
163	874.60	0.16	B1	204	1108.41	0.67	B3
164	875.20	81.95	A	205	1123.07	51.11	A
165	922.94	19.25	A	206	1123.09	1.06	B1
166	922.99	8.31	B1	207	1126.54	1.36	B3
167	924.00	17.87	B3	208	1128.27	22.12	B2
168	924.03	0.20	B2	209	1158.06	29.17	A
169	972.49	0.52	B3	210	1160.21	6.92	B3
170	972.96	5.57	B2	211	1160.56	7.78	B2
171	975.18	6.82	B1	212	1160.85	12.92	B1
172	975.20	84.85	A	213	1197.30	0.05	B2
173	1012.87	4.43	B3	214	1197.94	7.68	B3
174	1013.73	29.28	B2	215	1200.45	29.57	A
175	1014.36	77.78	A	216	1200.51	0.91	B1
176	1014.87	25.08	A	217	1221.12	11.96	B2
177	1015.16	6.85	B1	218	1221.92	25.33	A
178	1015.99	6.11	B2	219	1223.26	103.10	B1
179	1016.36	0.01	B3	220	1225.79	32.09	B3
180	1016.95	0.28	B1	221	1228.42	0.66	B3
181	1058.46	76.95	A	222	1230.88	64.97	A
182	1058.55	0.65	B1	223	1231.35	3.71	B1
183	1058.96	0.03	B3	224	1236.49	2.13	B1
184	1060.34	81.72	A	225	1236.92	0.06	B2
185	1060.51	5.80	B2	226	1238.71	18.02	B3
186	1061.46	0.44	B2	227	1239.01	51.19	A
187	1061.61	5.86	B3	228	1239.52	25.18	B2
188	1061.64	1.08	B1	229	1252.37	52.11	A
189	1073.29	97.34	A	230	1253.13	19.06	B1
190	1073.47	11.16	B2	231	1254.50	4.07	B3
191	1073.85	29.06	B3	232	1255.22	0.26	B2
192	1075.29	1.91	B1	233	1272.24	11.04	B3
193	1079.26	0.73	B1	234	1272.79	3.79	B2
194	1079.63	27.00	A	235	1276.59	0.09	B1
195	1082.15	2.43	B2	236	1277.84	6.94	A
196	1092.56	2.51	B3	237	1285.82	8.78	B2
197	1094.90	18.95	B3	238	1287.89	4.34	B3
198	1095.36	206.31	A	239	1289.95	43.49	A

240	1292.82	4.15	B1	281	1464.28	2.98	B3
241	1301.15	25.23	A	282	1465.66	0.08	B2
242	1302.68	25.34	B3	283	1465.76	3.44	B1
243	1303.83	1.22	B2	284	1466.36	35.72	A
244	1305.51	25.99	B1	285	1473.61	27.78	B2
245	1311.36	50.56	B3	286	1473.74	59.88	B3
246	1311.73	41.66	A	287	1475.42	17.19	A
247	1313.04	0.86	B2	288	1476.57	79.02	B1
248	1313.18	22.27	B1	289	1490.07	113.57	B3
249	1313.78	9.72	B1	290	1490.53	1000.00	A
250	1317.52	1.04	B2	291	1493.36	85.15	B1
251	1317.92	36.89	A	292	1493.70	113.51	B2
252	1319.30	1.76	B3	293	1596.11	22.37	B2
253	1322.06	38.08	A	294	1597.85	59.85	B1
254	1324.49	7.64	B2	295	1603.78	294.76	A
255	1325.19	7.84	B3	296	1604.28	60.65	B3
256	1330.70	4.11	B1	297	1655.27	148.09	A
257	1354.87	95.14	A	298	1656.68	30.08	B3
258	1355.10	0.44	B3	299	1681.68	4.59	B2
259	1355.17	3.29	B1	300	1687.70	4.19	B1
260	1355.50	0.01	B2	301	2950.00	197.99	A
261	1357.19	69.71	A	302	2950.04	0.53	B1
262	1358.08	4.40	B1	303	2950.70	87.46	B3
263	1358.17	1.54	B3	304	2950.77	27.59	B2
264	1359.50	11.89	B2	305	2966.32	56.45	B3
265	1398.92	15.46	A	306	2966.33	1.36	B2
266	1399.88	8.21	B3	307	2966.55	0.17	B1
267	1400.80	4.95	B2	308	2966.81	584.43	A
268	1401.13	22.28	B1	309	2979.45	72.15	A
269	1420.67	16.49	B2	310	2979.50	1.29	B1
270	1421.32	19.12	B1	311	2980.47	13.08	B2
271	1421.55	207.12	A	312	2980.69	22.97	B3
272	1421.58	19.25	B3	313	2992.56	31.51	A
273	1438.32	17.21	B3	314	2992.81	18.03	B1
274	1438.73	20.36	B2	315	2993.23	111.32	B2
275	1439.86	26.91	A	316	2993.52	0.87	B3
276	1442.40	1.11	B1	317	2995.76	12.93	B2
277	1457.94	18.02	B2	318	2996.02	0.21	B3
278	1460.83	2.88	B3	319	2996.54	4.99	B1
279	1461.39	106.60	A	320	2996.57	525.27	A
280	1463.81	1.19	B1	321	3008.89	116.07	B1

322	3008.96	114.95	A
323	3009.31	19.12	B2
324	3009.37	1.73	B3
325	3071.00	31.94	B2
326	3076.32	385.00	A
327	3080.59	14.86	B3
328	3081.71	161.65	B1
329	3186.74	0.13	B3
330	3186.87	165.26	A
331	3186.88	3.93	B1
332	3186.90	10.22	B2
333	3226.52	30.17	B2
334	3226.99	604.63	A
335	3230.52	63.81	B3
336	3232.11	47.84	B1
337	3281.82	92.58	A
338	3285.87	0.23	B3
339	3288.83	11.76	B2
340	3295.96	49.22	B1
341	3331.17	10.32	B3
342	3331.27	383.70	A
343	3331.62	39.48	B2
344	3333.72	131.97	B1
345	3530.29	12.19	B2
346	3531.31	36.79	A
347	3531.63	12.92	B3
348	3533.68	38.36	B1

Table S-6. Solid-state DFT IR-active modes with frequencies (cm^{-1}), intensities (km/mol), and mode symmetries for R-II full optimization.

Mode Label (ν)	Frequency (cm^{-1})	Intensity (km/mol)	Mode Symmetry	Mode Label (ν)	Frequency (cm^{-1})	Intensity (km/mol)	Mode Symmetry
6	34.22	0.22	B2	54	215.07	62.16	B3
7	40.40	0.55	B2	55	217.98	6.03	B1
8	41.01	0.96	B1	56	228.51	75.17	B2
10	50.40	0.14	B1	58	241.90	0.47	B3
11	51.88	0.11	B3	59	242.81	0.20	B2
12	58.73	2.29	B3	60	250.50	10.84	B1
13	59.75	1.76	B2	61	264.11	9.49	B2
14	63.46	1.32	B1	62	265.52	8.23	B3
15	69.91	16.74	B3	63	266.23	6.31	B1
17	77.69	0.52	B1	64	276.09	8.15	B1
18	82.77	20.72	B2	67	293.98	53.90	B2
20	86.40	1.21	B3	68	300.62	1.02	B3
21	93.39	12.72	B3	70	321.22	8.35	B3
23	95.67	16.63	B2	71	331.72	85.82	B2
24	97.52	1.98	B1	72	333.03	16.29	B1
25	104.28	21.31	B2	74	340.82	1.69	B2
26	106.41	0.94	B1	75	343.28	51.62	B3
28	118.16	0.04	B3	76	346.56	58.45	B1
29	119.21	45.56	B2	77	360.00	81.03	B3
30	120.09	0.25	B1	79	375.18	1.50	B1
32	131.51	9.26	B3	80	375.88	86.06	B2
33	132.43	0.21	B1	81	377.37	6.29	B2
35	138.92	13.07	B2	82	386.73	2.27	B1
36	139.97	6.97	B3	83	397.41	0.00	B3
37	141.72	0.07	B1	85	430.28	1.33	B1
39	147.61	1.43	B2	86	431.00	2.03	B2
40	148.66	18.35	B3	87	434.38	59.42	B3
41	160.39	5.16	B3	90	464.55	63.49	B3
42	166.43	1.80	B2	91	464.86	11.94	B1
43	167.88	28.96	B3	92	465.14	4.29	B2
44	168.71	33.64	B2	94	523.57	0.05	B1
46	172.33	0.01	B1	95	527.60	98.86	B2
47	177.08	0.02	B1	96	531.34	136.87	B3
49	182.66	11.25	B3	97	546.32	36.16	B1
51	199.46	85.96	B2	98	546.75	58.15	B2
52	201.17	10.14	B1	99	547.71	28.98	B3

101	581.73	24.20	B1	156	877.24	22.22	B2
102	583.10	187.96	B3	157	884.70	186.58	B2
104	588.70	1.23	B2	158	885.29	272.72	B1
106	593.78	12.06	B2	159	893.54	3.43	B3
107	596.07	615.58	B3	161	895.68	0.02	B2
108	599.72	4.57	B1	162	896.40	132.86	B1
109	652.21	44.32	B2	164	920.48	7.33	B3
110	652.28	48.66	B1	165	943.56	141.38	B2
112	652.84	8.78	B3	167	944.00	136.35	B1
113	659.73	550.21	B3	168	944.14	256.80	B3
114	660.10	15.10	B2	169	1003.87	16.19	B3
116	670.14	14.02	B1	170	1004.31	3.89	B2
117	681.15	77.78	B3	171	1005.44	0.88	B1
119	684.04	36.14	B2	174	1014.40	0.24	B3
120	686.58	175.47	B1	175	1017.33	366.81	B2
121	694.90	11.86	B3	176	1018.65	0.53	B1
123	709.42	7.71	B1	178	1019.94	110.80	B3
124	709.53	211.95	B2	179	1025.25	692.70	B1
126	716.79	23.31	B3	180	1025.73	21.27	B2
127	747.00	288.39	B2	181	1035.62	565.52	B2
128	749.43	121.12	B1	183	1037.34	89.74	B3
129	763.52	30.89	B3	184	1038.27	117.21	B1
131	766.59	83.05	B2	185	1045.40	649.26	B2
132	767.94	37.87	B1	187	1050.18	135.19	B1
133	777.33	15.28	B3	188	1052.12	137.67	B3
134	777.44	8.93	B2	189	1057.68	13.63	B2
136	778.60	0.40	B1	190	1057.73	15.66	B1
137	794.45	61.59	B2	192	1064.20	153.39	B3
138	794.82	86.34	B1	193	1070.77	150.61	B1
139	801.91	490.61	B3	194	1071.42	104.80	B2
142	816.94	362.59	B1	196	1072.70	3.47	B3
143	817.91	91.31	B2	197	1081.60	47.00	B3
144	819.54	234.60	B3	199	1085.68	28.49	B2
146	822.33	61.90	B3	200	1087.18	125.42	B1
147	822.54	61.81	B1	201	1094.97	125.46	B3
148	822.63	170.85	B2	203	1103.79	31.64	B2
149	863.42	27.61	B2	204	1104.15	61.88	B1
150	864.43	316.05	B3	205	1126.66	14.87	B3
152	868.69	6.93	B1	207	1128.34	437.81	B2
154	873.00	0.30	B3	208	1132.09	79.18	B1
155	876.72	50.35	B1	210	1168.44	158.96	B1

211	1169.28	142.91	B3	265	1393.24	57.83	B2
212	1169.36	101.10	B2	266	1393.89	40.40	B1
213	1204.63	140.54	B2	268	1394.48	20.52	B3
214	1205.28	52.15	B1	269	1412.70	0.74	B2
216	1208.54	14.52	B3	270	1412.71	0.58	B1
217	1209.33	0.82	B2	272	1414.22	117.22	B3
218	1209.59	61.92	B1	273	1427.81	122.24	B2
219	1213.58	46.63	B3	274	1428.24	0.00	B1
222	1223.36	92.81	B3	276	1431.73	25.96	B3
223	1228.81	5.43	B1	277	1453.35	22.85	B3
224	1229.78	9.01	B2	279	1458.61	310.75	B2
225	1244.37	0.07	B3	280	1458.87	112.73	B1
227	1249.76	287.88	B2	282	1466.23	228.22	B1
228	1253.08	59.53	B1	283	1466.42	656.53	B2
229	1261.17	3.70	B2	284	1469.46	55.63	B3
230	1261.21	18.63	B1	285	1473.84	1.90	B3
232	1268.32	126.03	B3	287	1478.17	465.01	B2
233	1272.97	162.23	B1	288	1480.38	150.17	B1
235	1278.11	142.67	B2	290	1490.80	11.98	B3
236	1279.49	159.12	B3	291	1492.82	120.65	B1
238	1298.91	25.29	B3	292	1493.35	12.87	B2
239	1299.67	60.49	B1	293	1591.95	51.34	B1
240	1300.87	29.98	B2	295	1596.67	559.96	B2
242	1308.86	74.30	B3	296	1599.64	38.50	B3
243	1313.71	28.98	B1	297	1628.26	695.09	B2
244	1313.97	7.53	B2	299	1631.45	2903.03	B1
245	1318.60	12.15	B3	300	1632.19	60.81	B3
247	1321.51	89.97	B2	301	2946.67	90.08	B3
248	1322.37	15.63	B1	303	2947.83	353.41	B1
249	1337.16	1.08	B3	304	2947.90	0.18	B2
251	1338.79	35.47	B1	305	2972.37	5234.97	B2
252	1338.87	0.66	B2	307	2976.29	80.39	B3
254	1343.52	31.81	B3	308	2978.69	52.43	B1
255	1351.19	9.75	B2	309	2992.47	1794.33	B2
256	1351.61	138.92	B1	310	2992.66	11.28	B1
257	1356.22	89.75	B1	312	2993.30	4513.95	B2
259	1356.83	6.64	B3	313	2994.16	351.73	B3
260	1357.26	0.00	B2	314	2994.74	3.52	B3
261	1360.56	62.46	B1	316	2999.30	0.47	B1
262	1360.71	0.27	B2	317	2999.40	21.70	B2
263	1367.57	11.84	B3	319	3001.81	131.85	B3

320	3012.70	398.54	B1
321	3016.00	18.86	B3
323	3016.88	92.03	B2
324	3019.21	329.21	B1
325	3047.65	239.85	B1
326	3047.99	154.75	B3
328	3048.03	48.57	B2
330	3061.90	7489.49	B1
331	3070.36	805.79	B2
332	3073.40	130.04	B3
334	3112.28	1252.65	B2
335	3113.31	5391.85	B3
336	3113.33	716.74	B1
337	3153.90	255.91	B2
338	3154.90	25.66	B3
340	3154.96	828.50	B1
342	3249.24	2503.04	B3
343	3274.06	4884.89	B2
344	3289.84	876.57	B1
345	3517.80	593.16	B2
347	3519.35	48.93	B1
348	3519.46	37.51	B3

Table S-7. Solid-state DFT Raman-active modes with frequencies (cm^{-1}), relative intensities (normalized to 1000), and mode symmetries for R-II full optimization at 78 K.

Mode Label (ν)	Frequency (cm^{-1})	Relative Intensity	Mode Symmetry	Mode Label (ν)	Frequency (cm^{-1})	Relative Intensity	Mode Symmetry
4	25.24	753.94	A	40	148.66	73.23	B3
5	33.09	9.49	A	41	160.39	3.65	B3
6	34.22	1.35	B2	42	166.43	0.64	B2
7	40.40	2.41	B2	43	167.88	4.91	B3
8	41.01	196.65	B1	44	168.71	2.82	B2
9	49.60	26.58	A	45	172.31	23.45	A
10	50.40	302.78	B1	46	172.33	3.44	B1
11	51.88	2.96	B3	47	177.08	2.21	B1
12	58.73	3.44	B3	48	180.91	14.18	A
13	59.75	13.72	B2	49	182.66	9.91	B3
14	63.46	18.10	B1	50	189.79	39.92	A
15	69.91	0.56	B3	51	199.46	6.62	B2
16	74.40	41.18	A	52	201.17	17.64	B1
17	77.69	39.58	B1	53	210.48	3.51	A
18	82.77	21.03	B2	54	215.07	5.33	B3
19	84.26	17.54	A	55	217.98	2.01	B1
20	86.40	3.00	B3	56	228.51	5.62	B2
21	93.39	0.89	B3	57	232.82	29.49	A
22	93.94	38.92	A	58	241.90	0.32	B3
23	95.67	2.53	B2	59	242.81	0.43	B2
24	97.52	1.21	B1	60	250.50	2.31	B1
25	104.28	0.66	B2	61	264.11	11.27	B2
26	106.41	26.14	B1	62	265.52	2.22	B3
27	109.28	54.49	A	63	266.23	19.45	B1
28	118.16	0.91	B3	64	276.09	0.53	B1
29	119.21	65.55	B2	65	277.54	7.85	A
30	120.09	2.99	B1	66	291.05	22.32	A
31	124.15	124.99	A	67	293.98	3.43	B2
32	131.51	43.53	B3	68	300.62	1.28	B3
33	132.43	32.44	B1	69	309.78	42.91	A
34	136.70	9.44	A	70	321.22	10.06	B3
35	138.92	30.44	B2	71	331.72	31.28	B2
36	139.97	3.21	B3	72	333.03	0.04	B1
37	141.72	0.32	B1	73	340.36	17.14	A
38	142.32	5.67	A	74	340.82	0.26	B2
39	147.61	613.56	B2	75	343.28	4.38	B3

76	346.56	21.50	B1	117	681.15	8.14	B3
77	360.00	8.49	B3	118	681.73	103.38	A
78	368.36	194.56	A	119	684.04	0.00	B2
79	375.18	12.61	B1	120	686.58	0.88	B1
80	375.88	1.10	B2	121	694.90	15.44	B3
81	377.37	1.32	B2	122	697.67	44.36	A
82	386.73	7.19	B1	123	709.42	37.77	B1
83	397.41	6.05	B3	124	709.53	0.03	B2
84	397.94	61.03	A	125	714.02	9.74	A
85	430.28	2.61	B1	126	716.79	0.03	B3
86	431.00	7.81	B2	127	747.00	4.15	B2
87	434.38	4.72	B3	128	749.43	23.95	B1
88	434.61	0.36	A	129	763.52	5.55	B3
89	463.68	35.03	A	130	763.63	12.76	A
90	464.55	33.81	B3	131	766.59	7.12	B2
91	464.86	2.00	B1	132	767.94	10.58	B1
92	465.14	0.02	B2	133	777.33	2.81	B3
93	523.17	49.22	A	134	777.44	3.95	B2
94	523.57	1.09	B1	135	778.08	6.54	A
95	527.60	24.75	B2	136	778.60	6.18	B1
96	531.34	0.44	B3	137	794.45	0.00	B2
97	546.32	23.02	B1	138	794.82	10.86	B1
98	546.75	2.13	B2	139	801.91	6.11	B3
99	547.71	10.75	B3	140	802.58	19.75	A
100	551.11	14.22	A	141	816.14	16.99	A
101	581.73	7.08	B1	142	816.94	4.23	B1
102	583.10	13.11	B3	143	817.91	0.37	B2
103	584.06	53.12	A	144	819.54	6.94	B3
104	588.70	1.12	B2	145	822.04	208.98	A
105	593.74	52.85	A	146	822.33	5.31	B3
106	593.78	3.57	B2	147	822.54	11.96	B1
107	596.07	0.01	B3	148	822.63	1.70	B2
108	599.72	12.93	B1	149	863.42	1.45	B2
109	652.21	3.70	B2	150	864.43	2.65	B3
110	652.28	2.09	B1	151	868.38	4.72	A
111	652.43	7.25	A	152	868.69	17.15	B1
112	652.84	0.04	B3	153	871.84	130.29	A
113	659.73	0.17	B3	154	873.00	0.19	B3
114	660.10	6.22	B2	155	876.72	2.78	B1
115	668.78	0.79	A	156	877.24	3.77	B2
116	670.14	0.73	B1	157	884.70	28.25	B2

158	885.29	0.20	B1	199	1085.68	0.34	B2
159	893.54	0.06	B3	200	1087.18	11.78	B1
160	893.92	5.19	A	201	1094.97	3.74	B3
161	895.68	0.16	B2	202	1095.00	41.31	A
162	896.40	7.99	B1	203	1103.79	0.44	B2
163	920.24	3.97	A	204	1104.15	3.48	B1
164	920.48	0.59	B3	205	1126.66	21.03	B3
165	943.56	1.52	B2	206	1126.67	28.66	A
166	943.87	110.27	A	207	1128.34	0.89	B2
167	944.00	0.01	B1	208	1132.09	1.68	B1
168	944.14	3.28	B3	209	1167.54	48.55	A
169	1003.87	9.78	B3	210	1168.44	1.08	B1
170	1004.31	0.00	B2	211	1169.28	27.81	B3
171	1005.44	1.45	B1	212	1169.36	25.17	B2
172	1005.54	72.65	A	213	1204.63	0.63	B2
173	1013.52	49.01	A	214	1205.28	0.02	B1
174	1014.40	3.73	B3	215	1207.57	58.86	A
175	1017.33	4.16	B2	216	1208.54	12.00	B3
176	1018.65	27.92	B1	217	1209.33	12.81	B2
177	1019.89	29.00	A	218	1209.59	3.77	B1
178	1019.94	1.26	B3	219	1213.58	0.36	B3
179	1025.25	0.77	B1	220	1214.58	18.73	A
180	1025.73	12.49	B2	221	1222.65	29.26	A
181	1035.62	0.58	B2	222	1223.36	2.44	B3
182	1037.13	239.09	A	223	1228.81	1.17	B1
183	1037.34	0.46	B3	224	1229.78	2.98	B2
184	1038.27	3.56	B1	225	1244.37	50.76	B3
185	1045.40	0.97	B2	226	1244.95	15.19	A
186	1049.82	66.77	A	227	1249.76	0.01	B2
187	1050.18	0.01	B1	228	1253.08	8.03	B1
188	1052.12	11.71	B3	229	1261.17	4.73	B2
189	1057.68	24.30	B2	230	1261.21	6.04	B1
190	1057.73	31.15	B1	231	1268.00	20.31	A
191	1063.99	40.41	A	232	1268.32	0.01	B3
192	1064.20	0.58	B3	233	1272.97	9.17	B1
193	1070.77	5.48	B1	234	1274.37	11.40	A
194	1071.42	1.81	B2	235	1278.11	3.53	B2
195	1072.11	244.68	A	236	1279.49	0.74	B3
196	1072.70	18.00	B3	237	1296.94	71.38	A
197	1081.60	6.00	B3	238	1298.91	5.11	B3
198	1082.75	38.71	A	239	1299.67	1.34	B1

240	1300.87	0.09	B2	281	1463.27	163.86	A
241	1307.90	7.76	A	282	1466.23	17.97	B1
242	1308.86	0.15	B3	283	1466.42	1.27	B2
243	1313.71	8.00	B1	284	1469.46	0.23	B3
244	1313.97	0.04	B2	285	1473.84	27.83	B3
245	1318.60	23.83	B3	286	1474.28	453.02	A
246	1319.04	13.00	A	287	1478.17	5.62	B2
247	1321.51	0.36	B2	288	1480.38	44.73	B1
248	1322.37	13.66	B1	289	1489.23	381.62	A
249	1337.16	8.78	B3	290	1490.80	43.45	B3
250	1337.68	37.82	A	291	1492.82	15.47	B1
251	1338.79	22.94	B1	292	1493.35	2.48	B2
252	1338.87	39.39	B2	293	1591.95	23.30	B1
253	1342.75	6.63	A	294	1592.02	242.18	A
254	1343.52	0.02	B3	295	1596.67	0.12	B2
255	1351.19	2.08	B2	296	1599.64	2.73	B3
256	1351.61	1.42	B1	297	1628.26	0.96	B2
257	1356.22	3.94	B1	298	1631.18	174.52	A
258	1356.56	108.56	A	299	1631.45	20.40	B1
259	1356.83	12.68	B3	300	1632.19	14.74	B3
260	1357.26	10.21	B2	301	2946.67	0.03	B3
261	1360.56	8.51	B1	302	2946.85	372.92	A
262	1360.71	13.76	B2	303	2947.83	6.77	B1
263	1367.57	1.00	B3	304	2947.90	15.28	B2
264	1368.26	25.23	A	305	2972.37	123.03	B2
265	1393.24	0.00	B2	306	2975.51	1000.00	A
266	1393.89	5.58	B1	307	2976.29	48.60	B3
267	1394.42	83.55	A	308	2978.69	21.32	B1
268	1394.48	2.69	B3	309	2992.47	4.27	B2
269	1412.70	19.25	B2	310	2992.66	122.69	B1
270	1412.71	1.42	B1	311	2992.91	422.09	A
271	1414.03	94.87	A	312	2993.30	1.04	B2
272	1414.22	0.92	B3	313	2994.16	12.68	B3
273	1427.81	28.95	B2	314	2994.74	157.81	B3
274	1428.24	4.77	B1	315	2994.75	21.49	A
275	1431.72	8.61	A	316	2999.30	0.52	B1
276	1431.73	1.05	B3	317	2999.40	2.97	B2
277	1453.35	0.84	B3	318	3001.59	115.86	A
278	1454.14	13.37	A	319	3001.81	28.61	B3
279	1458.61	4.48	B2	320	3012.70	19.61	B1
280	1458.87	8.72	B1	321	3016.00	9.67	B3

322	3016.13	163.81	A
323	3016.88	0.64	B2
324	3019.21	0.51	B1
325	3047.65	38.88	B1
326	3047.99	1.84	B3
327	3048.02	403.77	A
328	3048.03	66.65	B2
329	3060.84	428.18	A
330	3061.90	10.75	B1
331	3070.36	0.41	B2
332	3073.40	142.68	B3
333	3109.58	113.82	A
334	3112.28	0.20	B2
335	3113.31	38.63	B3
336	3113.33	152.49	B1
337	3153.90	0.35	B2
338	3154.90	55.89	B3
339	3154.96	177.67	A
340	3154.96	0.40	B1
341	3249.22	205.27	A
342	3249.24	65.51	B3
343	3274.06	41.03	B2
344	3289.84	124.31	B1
345	3517.80	5.85	B2
346	3519.28	93.30	A
347	3519.35	0.99	B1
348	3519.46	67.17	B3

Table S-8. Solid-state DFT IR-active modes with frequencies (cm^{-1}), intensities (km/mol), and mode symmetries for R-II fixed-lattice optimization.

Mode Label (ν)	Frequency (cm^{-1})	Intensity (km/mol)	Mode Symmetry	Mode Label (ν)	Frequency (cm^{-1})	Intensity (km/mol)	Mode Symmetry
6	32.74	0.04	B2	54	213.35	66.97	B3
7	39.50	0.94	B1	55	216.81	7.25	B1
8	40.06	0.67	B2	56	226.72	75.35	B2
10	50.35	0.23	B3	58	239.94	0.66	B3
11	51.72	0.29	B1	59	241.72	0.25	B2
12	58.49	2.51	B3	60	246.99	11.28	B1
13	59.07	1.80	B2	61	263.70	9.93	B2
14	61.89	0.83	B1	62	265.94	6.97	B1
15	68.11	16.98	B3	63	266.29	6.94	B3
17	75.00	0.35	B1	64	275.37	9.03	B1
18	79.61	20.58	B2	67	291.83	42.55	B2
20	84.87	1.12	B3	68	298.00	0.94	B3
21	91.29	12.16	B3	70	317.61	10.68	B3
23	93.81	12.21	B2	71	329.51	98.68	B2
24	95.19	1.59	B1	72	331.61	14.09	B1
25	104.22	18.04	B2	73	338.17	1.90	B2
26	108.14	1.61	B1	75	343.11	58.00	B3
28	116.66	0.49	B3	76	345.11	53.97	B1
29	116.95	53.61	B2	77	357.69	55.78	B3
30	117.05	0.30	B1	79	373.03	0.90	B1
32	129.55	0.09	B1	80	373.71	67.30	B2
33	130.26	7.46	B3	81	377.18	15.28	B2
35	135.51	7.39	B2	82	383.63	5.50	B1
36	137.22	9.46	B3	83	395.66	0.16	B3
38	140.78	0.11	B1	85	427.77	0.91	B1
39	145.78	1.44	B2	86	428.42	0.94	B2
40	146.36	12.57	B3	87	430.35	66.30	B3
41	158.75	8.23	B3	90	461.36	53.49	B3
42	165.39	30.51	B2	91	462.05	2.51	B2
43	165.78	21.86	B3	92	462.28	12.37	B1
44	166.92	7.36	B2	93	520.91	0.00	B1
46	170.01	0.14	B1	95	525.17	104.66	B2
47	174.95	0.02	B1	96	529.13	130.70	B3
49	182.17	13.70	B3	97	544.40	47.08	B2
51	197.61	75.33	B2	98	544.80	37.84	B1
52	199.75	9.53	B1	99	545.97	15.59	B3

101	583.84	13.59	B1	156	875.69	0.11	B2
102	583.93	43.50	B3	157	878.22	157.41	B2
104	588.02	7.85	B2	158	879.54	137.91	B1
106	597.11	2.88	B2	159	894.36	6.01	B3
107	598.47	746.60	B3	161	895.11	8.79	B2
108	600.78	8.74	B1	162	896.04	52.48	B1
109	652.71	48.60	B1	164	913.13	6.81	B3
111	652.94	47.03	B2	165	943.16	156.05	B2
112	653.35	34.92	B3	166	944.09	274.92	B3
113	661.42	563.98	B3	168	944.44	137.01	B1
114	661.83	5.04	B2	169	1003.35	10.75	B2
116	671.22	16.35	B1	170	1003.59	16.08	B3
117	677.65	94.15	B3	171	1004.44	3.00	B1
119	683.16	49.15	B2	174	1013.87	0.84	B3
120	685.65	167.65	B1	175	1018.31	354.78	B2
121	690.18	4.34	B3	176	1019.79	0.77	B1
123	707.98	226.88	B2	177	1019.94	106.64	B3
124	708.16	5.95	B1	179	1024.42	691.72	B1
126	714.38	23.52	B3	180	1024.77	28.20	B2
127	743.04	254.26	B2	181	1034.74	602.28	B2
128	744.95	130.33	B1	183	1037.18	85.03	B3
130	760.87	27.11	B3	184	1037.88	114.21	B1
131	763.48	78.10	B2	185	1043.90	614.10	B2
132	764.79	59.16	B1	186	1048.47	109.61	B1
133	778.04	5.47	B3	188	1051.29	145.30	B3
134	778.14	9.21	B2	189	1058.08	28.97	B2
136	779.03	0.85	B1	190	1058.30	7.33	B1
137	787.66	88.70	B1	192	1064.49	172.97	B3
138	787.72	23.43	B2	194	1071.44	168.95	B1
139	794.05	545.55	B3	195	1071.87	112.30	B2
141	814.48	380.12	B1	196	1073.41	3.30	B3
143	814.95	106.40	B2	197	1081.53	33.65	B3
144	817.23	254.01	B3	199	1085.85	35.43	B2
146	822.04	31.34	B1	200	1087.34	133.38	B1
147	822.17	0.68	B3	201	1095.71	113.74	B3
148	822.20	211.51	B2	203	1103.48	33.43	B2
149	864.37	47.15	B2	204	1104.26	53.28	B1
150	866.10	285.16	B3	206	1124.54	13.55	B3
151	869.62	137.94	B1	207	1126.31	466.94	B2
153	872.59	12.75	B3	208	1129.18	73.98	B1
155	874.57	122.68	B1	210	1168.36	153.52	B1

211	1169.27	108.76	B2	265	1392.75	61.82	B2
212	1169.63	134.10	B3	266	1394.14	40.10	B1
213	1203.56	146.88	B2	268	1394.83	31.98	B3
214	1204.21	57.00	B1	269	1412.63	1.65	B1
216	1207.41	9.48	B3	270	1412.92	0.88	B2
217	1208.64	6.17	B2	271	1414.06	99.65	B3
218	1208.92	59.37	B1	273	1425.43	112.78	B2
219	1212.66	46.79	B3	274	1425.92	0.24	B1
222	1223.14	99.95	B3	276	1429.00	24.23	B3
223	1228.96	5.04	B1	277	1450.43	24.47	B3
224	1230.06	11.34	B2	279	1455.22	232.93	B2
225	1244.72	0.78	B3	280	1456.25	66.27	B1
227	1251.12	273.73	B2	282	1464.57	260.97	B1
228	1254.20	57.10	B1	283	1465.88	740.87	B2
229	1262.09	5.25	B2	284	1467.52	25.71	B3
230	1262.27	18.16	B1	285	1470.68	15.59	B3
232	1269.31	126.42	B3	287	1475.95	433.80	B2
233	1272.83	177.22	B1	288	1478.21	152.50	B1
235	1278.48	164.74	B2	290	1488.82	11.65	B3
236	1279.53	146.03	B3	291	1489.53	139.64	B1
238	1299.21	30.76	B3	292	1490.46	4.97	B2
239	1301.07	59.30	B1	293	1591.96	46.99	B1
240	1301.21	17.87	B2	295	1596.36	623.06	B2
242	1309.90	52.57	B3	296	1599.53	28.53	B3
243	1315.39	23.06	B1	297	1626.52	697.91	B2
244	1315.51	25.45	B2	299	1630.17	2889.39	B1
246	1321.09	11.88	B3	300	1631.09	40.76	B3
247	1321.82	89.42	B2	301	2949.03	57.04	B3
248	1323.52	18.95	B1	303	2949.82	318.38	B1
250	1338.62	1.82	B3	304	2949.96	0.53	B2
251	1340.02	1.44	B2	305	2974.75	1380.30	B2
252	1340.07	42.30	B1	307	2977.68	13.60	B1
254	1344.10	26.61	B3	308	2977.74	5.95	B3
255	1349.49	11.40	B2	309	2992.81	16.55	B1
256	1350.77	91.51	B1	310	2992.92	8.74	B2
257	1355.55	1.37	B2	311	2993.82	25.87	B3
258	1356.14	9.59	B3	312	2994.45	161.17	B2
259	1356.16	56.57	B1	314	2995.29	0.01	B3
261	1360.24	104.54	B1	316	2995.48	11.08	B1
262	1360.84	0.02	B2	317	3007.00	9940.74	B2
263	1366.80	12.43	B3	319	3010.97	243.45	B3

320	3013.23	6.96	B2
322	3013.37	311.23	B3
323	3013.58	6.65	B1
324	3032.01	841.87	B1
326	3043.75	111.07	B2
327	3043.76	131.25	B3
328	3044.20	47.19	B1
330	3075.90	7309.11	B1
331	3084.09	754.85	B2
332	3086.96	86.60	B3
334	3128.52	1368.46	B2
335	3129.33	651.88	B1
336	3130.07	4871.64	B3
337	3154.42	247.52	B2
338	3154.55	835.59	B1
340	3155.83	61.94	B3
341	3266.48	2343.80	B3
343	3289.84	4857.17	B2
344	3305.10	842.86	B1
345	3516.17	608.14	B2
347	3517.13	44.43	B1
348	3517.96	24.23	B3

Table S-9. Solid-state DFT Raman-active modes with frequencies (cm^{-1}), relative intensities (normalized to 1000), and mode symmetries for R-II fixed-lattice optimization at 295 K.

Mode Label (ν)	Frequency (cm^{-1})	Relative Intensity	Mode Symmetry	Mode Label (ν)	Frequency (cm^{-1})	Relative Intensity	Mode Symmetry
4	24.01	1000.00	A	40	146.36	44.04	B3
5	31.66	19.86	A	41	158.75	4.33	B3
6	32.74	2.83	B2	42	165.39	0.77	B2
7	39.50	198.99	B1	43	165.78	3.20	B3
8	40.06	4.41	B2	44	166.92	0.51	B2
9	47.47	27.26	A	45	168.89	19.13	A
10	50.35	5.31	B3	46	170.01	5.07	B1
11	51.72	510.68	B1	47	174.95	0.80	B1
12	58.49	8.96	B3	48	179.98	11.67	A
13	59.07	6.75	B2	49	182.17	8.03	B3
14	61.89	12.04	B1	50	189.81	31.90	A
15	68.11	0.78	B3	51	197.61	3.99	B2
16	74.39	25.22	A	52	199.75	9.69	B1
17	75.00	38.15	B1	53	210.54	3.02	A
18	79.61	21.68	B2	54	213.35	3.52	B3
19	80.19	32.91	A	55	216.81	1.64	B1
20	84.87	2.52	B3	56	226.72	4.44	B2
21	91.29	1.54	B3	57	230.68	15.86	A
22	93.34	20.57	A	58	239.94	0.61	B3
23	93.81	2.61	B2	59	241.72	0.12	B2
24	95.19	4.72	B1	60	246.99	2.80	B1
25	104.22	0.91	B2	61	263.70	6.96	B2
26	108.14	24.92	B1	62	265.94	11.63	B1
27	109.17	77.10	A	63	266.29	1.99	B3
28	116.66	1.17	B3	64	275.37	0.57	B1
29	116.95	62.60	B2	65	276.49	4.98	A
30	117.05	1.83	B1	66	288.99	11.98	A
31	124.29	113.73	A	67	291.83	3.10	B2
32	129.55	35.66	B1	68	298.00	1.24	B3
33	130.26	36.28	B3	69	308.13	23.65	A
34	133.55	8.55	A	70	317.61	6.28	B3
35	135.51	55.61	B2	71	329.51	15.94	B2
36	137.22	0.21	B3	72	331.61	0.01	B1
37	140.09	5.61	A	73	338.17	0.41	B2
38	140.78	2.93	B1	74	340.52	11.41	A
39	145.78	476.69	B2	75	343.11	2.24	B3

76	345.11	13.51	B1	117	677.65	7.31	B3
77	357.69	3.86	B3	118	678.01	33.68	A
78	363.99	109.46	A	119	683.16	0.02	B2
79	373.03	6.16	B1	120	685.65	0.58	B1
80	373.71	1.04	B2	121	690.18	4.88	B3
81	377.18	0.54	B2	122	693.20	33.49	A
82	383.63	2.26	B1	123	707.98	0.05	B2
83	395.66	3.98	B3	124	708.16	17.86	B1
84	396.29	27.60	A	125	712.29	4.38	A
85	427.77	1.32	B1	126	714.38	0.02	B3
86	428.42	4.87	B2	127	743.04	2.08	B2
87	430.35	2.71	B3	128	744.95	13.31	B1
88	430.76	0.44	A	129	760.19	5.24	A
89	461.21	19.26	A	130	760.87	2.03	B3
90	461.36	15.52	B3	131	763.48	3.73	B2
91	462.05	0.00	B2	132	764.79	3.20	B1
92	462.28	0.74	B1	133	778.04	1.60	B3
93	520.91	0.52	B1	134	778.14	1.69	B2
94	521.29	24.07	A	135	778.53	2.99	A
95	525.17	11.73	B2	136	779.03	3.54	B1
96	529.13	0.26	B3	137	787.66	3.93	B1
97	544.40	0.66	B2	138	787.72	0.01	B2
98	544.80	10.69	B1	139	794.05	2.02	B3
99	545.97	6.01	B3	140	794.42	6.97	A
100	549.52	8.07	A	141	814.48	3.29	B1
101	583.84	2.32	B1	142	814.69	0.81	A
102	583.93	6.45	B3	143	814.95	0.04	B2
103	584.59	30.18	A	144	817.23	5.91	B3
104	588.02	1.35	B2	145	821.81	104.85	A
105	596.70	17.87	A	146	822.04	6.06	B1
106	597.11	0.64	B2	147	822.17	0.64	B3
107	598.47	0.34	B3	148	822.20	0.74	B2
108	600.78	8.36	B1	149	864.37	0.02	B2
109	652.71	1.40	B1	150	866.10	0.72	B3
110	652.87	3.90	A	151	869.62	9.90	B1
111	652.94	2.09	B2	152	870.22	23.10	A
112	653.35	0.13	B3	153	872.59	0.18	B3
113	661.42	0.17	B3	154	872.64	38.27	A
114	661.83	1.83	B2	155	874.57	0.00	B1
115	669.17	0.31	A	156	875.69	7.87	B2
116	671.22	0.30	B1	157	878.22	5.40	B2

158	879.54	0.20	B1	199	1085.85	0.10	B2
159	894.36	0.00	B3	200	1087.34	4.60	B1
160	894.87	1.67	A	201	1095.71	1.63	B3
161	895.11	1.06	B2	202	1096.53	19.14	A
162	896.04	2.99	B1	203	1103.48	0.12	B2
163	912.93	2.35	A	204	1104.26	1.60	B1
164	913.13	0.21	B3	205	1124.20	12.03	A
165	943.16	0.50	B2	206	1124.54	10.19	B3
166	944.09	0.89	B3	207	1126.31	0.40	B2
167	944.13	52.63	A	208	1129.18	0.70	B1
168	944.44	0.01	B1	209	1167.66	22.50	A
169	1003.35	0.00	B2	210	1168.36	0.30	B1
170	1003.59	4.33	B3	211	1169.27	9.68	B2
171	1004.44	0.39	B1	212	1169.63	13.14	B3
172	1004.53	37.88	A	213	1203.56	0.06	B2
173	1012.94	24.83	A	214	1204.21	0.01	B1
174	1013.87	1.92	B3	215	1206.78	25.73	A
175	1018.31	1.37	B2	216	1207.41	6.32	B3
176	1019.79	12.06	B1	217	1208.64	5.73	B2
177	1019.94	0.73	B3	218	1208.92	1.23	B1
178	1020.04	11.79	A	219	1212.66	0.15	B3
179	1024.42	0.40	B1	220	1214.04	9.29	A
180	1024.77	6.19	B2	221	1222.40	13.07	A
181	1034.74	0.19	B2	222	1223.14	1.22	B3
182	1036.98	104.92	A	223	1228.96	0.42	B1
183	1037.18	0.16	B3	224	1230.06	1.08	B2
184	1037.88	1.34	B1	225	1244.72	23.41	B3
185	1043.90	0.47	B2	226	1245.27	6.32	A
186	1048.47	0.02	B1	227	1251.12	0.02	B2
187	1048.78	30.26	A	228	1254.20	4.23	B1
188	1051.29	5.01	B3	229	1262.09	2.50	B2
189	1058.08	11.54	B2	230	1262.27	2.85	B1
190	1058.30	13.95	B1	231	1268.67	10.67	A
191	1064.31	16.66	A	232	1269.31	0.04	B3
192	1064.49	0.36	B3	233	1272.83	3.95	B1
193	1071.37	115.14	A	234	1273.98	5.20	A
194	1071.44	1.61	B1	235	1278.48	1.10	B2
195	1071.87	0.53	B2	236	1279.53	0.19	B3
196	1073.41	8.79	B3	237	1298.36	33.14	A
197	1081.53	2.73	B3	238	1299.21	2.37	B3
198	1082.84	15.76	A	239	1301.07	0.63	B1

240	1301.21	0.02	B2	281	1462.28	77.52	A
241	1309.41	2.59	A	282	1464.57	4.86	B1
242	1309.90	0.11	B3	283	1465.88	0.62	B2
243	1315.39	2.47	B1	284	1467.52	2.91	B3
244	1315.51	0.00	B2	285	1470.68	9.45	B3
245	1320.80	5.71	A	286	1471.39	181.39	A
246	1321.09	11.28	B3	287	1475.95	2.05	B2
247	1321.82	0.01	B2	288	1478.21	14.80	B1
248	1323.52	7.06	B1	289	1486.47	204.15	A
249	1338.49	20.56	A	290	1488.82	20.46	B3
250	1338.62	3.64	B3	291	1489.53	6.63	B1
251	1340.02	13.84	B2	292	1490.46	0.75	B2
252	1340.07	9.39	B1	293	1591.96	8.19	B1
253	1343.46	3.06	A	294	1592.00	115.75	A
254	1344.10	0.06	B3	295	1596.36	0.03	B2
255	1349.49	4.96	B2	296	1599.53	1.12	B3
256	1350.77	0.94	B1	297	1626.52	0.21	B2
257	1355.55	6.17	B2	298	1629.91	84.08	A
258	1356.14	5.72	B3	299	1630.17	7.64	B1
259	1356.16	2.72	B1	300	1631.09	6.85	B3
260	1356.37	40.18	A	301	2949.03	0.03	B3
261	1360.24	2.40	B1	302	2949.37	189.34	A
262	1360.84	3.02	B2	303	2949.82	3.39	B1
263	1366.80	0.35	B3	304	2949.96	7.31	B2
264	1367.22	13.11	A	305	2974.75	40.77	B2
265	1392.75	0.22	B2	306	2976.87	249.41	A
266	1394.14	1.36	B1	307	2977.68	13.09	B1
267	1394.68	31.22	A	308	2977.74	15.73	B3
268	1394.83	1.27	B3	309	2992.81	49.38	B1
269	1412.63	0.52	B1	310	2992.92	0.68	B2
270	1412.92	9.02	B2	311	2993.82	31.52	B3
271	1414.06	0.39	B3	312	2994.45	1.00	B2
272	1414.10	50.16	A	313	2994.95	164.59	A
273	1425.43	12.78	B2	314	2995.29	67.91	B3
274	1425.92	1.74	B1	315	2995.33	74.10	A
275	1428.97	3.31	A	316	2995.48	0.04	B1
276	1429.00	0.66	B3	317	3007.00	16.22	B2
277	1450.43	0.58	B3	318	3009.33	102.13	A
278	1450.65	4.34	A	319	3010.97	0.65	B3
279	1455.22	1.87	B2	320	3013.23	1.11	B2
280	1456.25	4.38	B1	321	3013.23	167.45	A

322	3013.37	1.53	B3
323	3013.58	7.20	B1
324	3032.01	8.27	B1
325	3043.48	182.75	A
326	3043.75	29.21	B2
327	3043.76	1.12	B3
328	3044.20	13.93	B1
329	3075.44	196.23	A
330	3075.90	2.79	B1
331	3084.09	0.01	B2
332	3086.96	65.03	B3
333	3126.44	62.66	A
334	3128.52	0.00	B2
335	3129.33	69.54	B1
336	3130.07	19.96	B3
337	3154.42	0.04	B2
338	3154.55	1.18	B1
339	3155.58	75.83	A
340	3155.83	22.38	B3
341	3266.48	30.24	B3
342	3266.54	96.03	A
343	3289.84	17.76	B2
344	3305.10	55.75	B1
345	3516.17	1.98	B2
346	3517.02	41.97	A
347	3517.13	0.20	B1
348	3517.96	32.32	B3

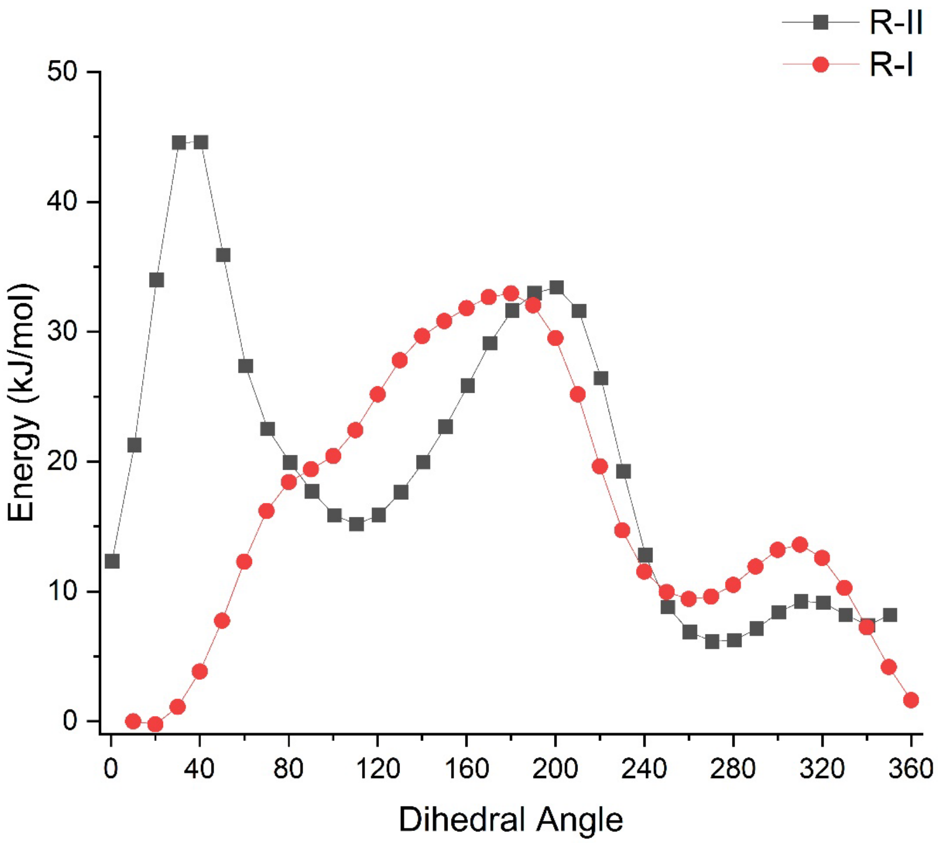

Figure S-6. Relative energy versus dihedral angle (O1-C1-N1-C6) curves for R-I and R-II.

Table S-10. Coordinates (angstroms) for R-I final conformation from Gaussian simulations.

Atom Type	X	Y	Z
H	-0.121565	1.803718	0.371693
O	0.544470	2.525743	0.318756
C	1.698626	2.158644	1.047786
C	2.237840	0.763651	0.737356
C	2.169846	0.382765	-0.746266
C	1.795935	-1.116767	-0.727626
O	1.459198	-0.234116	1.452107
O	3.448174	0.596058	-1.357135
O	2.960756	-1.916004	-0.642667
H	1.538102	2.218613	2.141740
H	3.678071	-1.355967	-1.011350
H	1.170952	-1.412134	-1.589904
H	3.300993	0.880089	-2.274752
H	2.479603	2.891099	0.786883
H	1.205433	-2.240976	1.041915
H	1.388085	0.977403	-1.240351
H	3.282439	0.688199	1.086355
C	1.022349	-1.252232	0.592979
N	-0.435849	-1.159050	0.412518
C	-1.381287	-2.118949	0.569194
N	-2.579954	-1.626874	0.287853
C	-2.308230	-0.326752	-0.040346
C	-3.357305	0.670308	-0.425517
N	-4.608098	0.131164	-0.433043
H	-5.395693	0.725410	-0.661423
H	-1.149924	-3.132962	0.884419
N	-1.014519	-0.004524	0.021542
O	-3.091433	1.834413	-0.699834
H	-4.743528	-0.840263	-0.172483

Table S-11. Experimental and simulated peak positions (cm^{-1}) in the terahertz spectrum of R-II.

Exp. 295 K (cm^{-1})	Exp. 20 K (cm^{-1})	Simulated (cm^{-1})
37	39	40.40, 41.01
53	57	58.73
56	60	59.75
61	64	63.46
67	69	69.91
77	81	82.77
88	93	93.39, 95.67
94	97	97.52
-	105	104.28
113	119	119.21
-	133	131.51
-	139	138.92, 139.97

Table S-12. Experimental and simulated peak positions (cm^{-1}) in the Raman spectrum of R-II.

Exp. 295 K (cm^{-1})	Exp. 78 K (cm^{-1})	Simulated (cm^{-1})
25	27	25.24
32	34	33.09
-	42	41.01
47	48	50.40
61	60	59.75
-	73	74.40
-	76	77.69
-	84	82.77, 84.26
-	92	93.94
107	110	109.28
118	121	124.15
144	150	147.61
